# Recommendations for the treatment of rheumatoid arthritis in Saudi Arabia: adolopment of the 2021 American College of Rheumatology guidelines

**DOI:** 10.1186/s41927-022-00301-y

**Published:** 2022-11-23

**Authors:** Mohammed A. Omair, Hanan Al Rayes, Joanne Khabsa, Sally Yaacoub, Sultana Abdulaziz, Ghada A. Al Janobi, Abdulaziz Al Khalaf, Bader Al Mehmadi, Mahasin Al Nassar, Faisal AlBalawi, Abdullah S. AlFurayj, Ahmed Hamdan Al-Jedai, Haya Mohammed Almalag, Hajer Yousef Almudaiheem, Ali AlRehaily, Mohammed A. Attar, Lina El Kibbi, Hussein Halabi, Manal Hasan, Jasvinder A. Singh, Liana Fraenkel, Elie A. Akl

**Affiliations:** 1 Rheumatology Unit, Department of Medicine, King Saud University, PO Box 2925, Riyadh, 11461 Saudi Arabia; 2 Division of Rheumatology, Department of Internal Medicine, Prince Sultan Military Medical City, Riyadh, Saudi Arabia; 3 AUB Grade Center, American University of Beirut Medical Center, Beirut, Lebanon; 4 Clinical Research Institute, American University of Beirut Medical Center, Beirut, Lebanon; 5 Division of Rheumatology, Department of Medicine, King Fahad Hospital, Jeddah, Saudi Arabia; 6 Rheumatology Unit, Department of Medicine, Qatif Central Hospital, Qatif, Saudi Arabia; 7 Division of Rheumatology, Department of Medicine, College of Medicine, Majmaah University, Al-Majmaah, 11952 Saudi Arabia; 8 Department of Obstetrics and Gynecology, King Saud University, Riyadh, Saudi Arabia; 9 Section of Rheumatology, Department of Internal Medicine, King Fahad Medical City, Riyadh, Saudi Arabia; 10 Rheumatology Unit, Department of Medicine, Buraidah Central Hospital, B.C.H, Buraidah, Qassim Saudi Arabia; 11 Deputyship of Therapeutic Affairs, Ministry of Health, Riyadh, Saudi Arabia; 12 College of Medicine, Alfaisal University, Riyadh, Saudi Arabia; 13 Department of Clinical Pharmacy, College of Pharmacy, King Saud University, Riyadh, Saudi Arabia; 14 Department of Medicine, Section of Rheumatology, Security Forces Hospital Program, Riyadh, Saudi Arabia; 15 Division of Rheumatology, Department of Medicine, Al Hada Armed Forces Hospital, Taif, Saudi Arabia; 16 Division of Rheumatology, Department of Internal Medicine, Specialized Medical Center, Riyadh, Saudi Arabia; 17 Section of Rheumatology, Department of Internal Medicine, King Faisal Specialist Hospital and Research Center-Jeddah, Jeddah, Saudi Arabia; 18 Division of Rheumatology, Department of Internal Medicine, Imam Abdulrahman Bin Faisal University, Dammam, Saudi Arabia; 19 Medicine Service, VA Medical Center, 700 19th St S, Birmingham, AL 35233 USA; 20 Department of Medicine at the School of Medicine, University of Alabama at Birmingham (UAB), 510 20th Street S, Birmingham, AL 35294-0022 USA; 21 Department of Epidemiology at the UAB School of Public Health, Ryals Public Health Building, 1665 University Blvd, Birmingham, AL 35294-0022 USA; 22 Berkshire Medical Center, Pittsfield, MA USA; 23 Yale University School of Medicine, New Haven, CT USA; 24 Department of Internal Medicine, American University of Beirut, Beirut, Lebanon; 25 Department of Health Research Methods, Evidence, and Impact (HEI), McMaster University, Hamilton, ON Canada

**Keywords:** Adaptation, Adolopment, Contextualization, Rheumatoid arthritis, American College of Rheumatology, Kingdom of Saudi Arabia, Glucocorticoids, Disease modifying anti-rheumatic drugs

## Abstract

**Background:**

The 2021 American College of Rheumatology (ACR) rheumatoid arthritis (RA) guideline considers the specific context of the United States which differs from that of Saudi Arabia in many aspects that may impact recommendations. The objective of this project was to adapt a set of prioritized recommendations from the 2021 ACR guideline for the treatment of rheumatoid arthritis RA for the context of Saudi Arabia, by the Saudi Society for Rheumatology (SSR).

**Methods:**

The process followed the GRADE-ADOLOPMENT methodology, and the reporting adhered to the RIGHT-Ad@pt checklist. Working groups included a coordination group and a 19-member panel representing different stakeholder groups. The Evidence to Decision (EtD) tables included evidence on health effects from the source guideline and contextual information from the Saudi setting.

**Results:**

The panel prioritized and adapted five recommendations from the source guideline. The process led to modifying two out of the five prioritized recommendations, all listed here. In naive patients with low disease activity, methotrexate (MTX) is conditionally recommended over sulfasalazine (SSZ) (modified direction); hydroxychloroquine (HCQ) is conditionally recommended over SSZ (unmodified). Initiation of csDMARDs with short-term glucocorticoids is conditionally recommended over csDMARDs alone in naive patients with moderate to high disease activity (modified direction). Switch to subcutaneous MTX is conditionally recommended over addition/switch to alternative DMARD(s) in patients taking oral MTX who are not at target (unmodified). Discontinuation of MTX is conditionally recommended over gradual discontinuation of the bDMARD or tsDMARD for patients taking MTX plus a bDMARD or tsDMARD who wish to discontinue a DMARD (unmodified).

**Conclusion:**

Rheumatologists practicing in Saudi Arabia can use the adoloped recommendations generated by this project while adopting the rest of the recommendations from the 2021 ACR guidelines.

**Supplementary Information:**

The online version contains supplementary material available at 10.1186/s41927-022-00301-y.

## Background

The global prevalence of rheumatoid arthritis (RA) is estimated to be 460 per 100,000 persons [1]. It remains a significant cause of disability and reduced quality of life in Saudi Arabia [2] and globally [3]. Early diagnosis, implementation of treat-to-target strategies, and the availability of highly effective disease-modifying anti-rheumatic drugs (DMARDs) have led to a dramatic improvement in disease-related outcomes [4].

Treatment guidelines for RA have been developed by well-established societies such as the American College of Rheumatology (ACR) and the European League Against Rheumatism (EULAR) [5, 6]. Recently, the ACR published its 2021 update of the guideline. They included 44 recommendations addressing questions on treatment with DMARDs, use of glucocorticoids, and use of DMARDs in certain high-risk populations [6].

The 2021 ACR RA recommendations consider the specific context of the United States (U.S.) which differs from that of Saudi Arabia in many aspects that may impact recommendations, e.g., costs, values, and preferences. Notably, the Saudi health system currently consists of several sectors that report to different authorities and have individualized health resources such as infusion units and medication formulary. Healthcare is free for National civilians through the Ministry of Health (MoH) and university institutions. In contrast, the military staff is followed in their respective military hospitals. On the other hand, expatriates can only access hospitals of the private sector and business centers of governmental institutions through their insurance plans.

Developing new guidelines is very time- and resource-intensive. A more reasonable alternative is to adapt the recommendations for the local context to ensure applicability and subsequent uptake [7]. The objective of this project was to adapt a set of prioritized recommendations from the 2021 ACR guideline for the treatment of RA, the 'source guideline,' for the context of Saudi Arabia. The resulting guidelines will be disseminated through different health sectors and used by policymakers for the 2030 health sector transformation.

## Methods

### Target population, end-users, and setting

Similar to the source guidelines, the target population are RA patients fulfilling the 2010 ACR/EULAR classification criteria [8]. In Saudi Arabia RA patients are exclusively managed by rheumatologists, who constitute the target audience of this work. Additional important stakeholders include patients, pharmacists, policymakers, and individuals in charge of coverage decisions in the private and public sectors.

### Guideline adaptation group

This project was a collaborative effort between the Saudi Society for Rheumatology (SSR), ACR, and the American University of Beirut (AUB) GRADE Center. The SSR was established in 2010 to improve education, research, and patient care in rheumatology. Working groups included a coordination group and a guideline panel.

The coordination group included two content experts (MAO, HAR) and three methodologists (EAA, JK, and SY). Its primary responsibilities included oversight of the work, designing the process and methodology, coordinating the prioritization of questions, summarizing findings for the panel through the preparation of Evidence to Decision (EtD) tables, and coordinating panel meetings.

The panel comprised 19 members representing the following stakeholder groups: 15 rheumatologists, one pharmacist, one patient representative, and two policymakers. The panel was balanced in gender, Saudi regions, and type of practice (governmental and private). It also included two international experts: the chair of the source guidelines (LF) and the chair of the 2015 ACR guideline for treating rheumatoid arthritis (JAS). Additional file 1 presents the list of panelists with their affiliation, stakeholder group, and COI declaration. The panel was co-chaired by a rheumatologist (MAO) and a methodologist (EAA). The first-panel meeting provided introductory training comprising a review of the methodology and a "mock" recommendation adaptation exercise.

The panel was involved in prioritizing questions and outcomes, providing relevant newly published studies, providing relevant contextual information, and participating in the final meeting in formulating the final recommendations.

### Adaptation methodology

For developing these guidelines, we followed the Grading of Recommendations Assessment, Development and Evaluation (GRADE)-ADOLOPMENT methodology [9] and the GIN-McMaster checklist for Guideline Development [10]. GRADE-ADOLOPMENT combines the advantages of adoption, adaptation, and de novo guideline development and is based on three cornerstones: (1) identifying credible and relevant existing guidelines; (2) developing GRADE EtD tables for each of the recommendations [11]; and (3) deciding on adoption, adaptation or de novo development for each of the recommendations [9]. In addition, we followed the recently published RIGHT-Ad@pt checklist for reporting adapted guidelines [12] for reporting these guidelines.

### Source guideline

The SSR selected the 2021 ACR guideline for the treatment of RA as the source guideline. The ACR guidelines follow rigorous methods for guideline development, including generation of population, intervention, comparator, and outcomes (PICO) questions, selection of critical outcomes, systematic literature review, and conflict of interest (COI) management. Although the ACR guidelines did not address all contextual factors included in GRADE EtD tables, they addressed patient values and preferences, cost-effectiveness studies, and cost information [6].

### Key questions

We used a structured and standardized process to prioritize five recommendations from the ACR guidelines. First, the coordination group asked the panelists to rate the priority of each of the original ACR recommendations based on whether the recommended intervention differed in the Saudi Arabian context (from the U.S. context) in terms of feasibility, acceptability, resource use, impact on equity and current practice. Additional file 2 presents the prioritized questions corresponding to the prioritized recommendation statements.

### Evidence synthesis

For the evidence on health effects, and as the adaptation project was launched shortly following the publication of the source guideline, we opted to use the evidence reports as published by the source guideline (search date: December 11, 2019) and did not formally update the systematic literature review for the adaptation process. However, we solicited panelists to share any newly published relevant studies. Two methodologist members of the coordination group (SY and JK) assessed the eligibility of these studies in duplicate and independently. For eligible studies, the two methodologists abstracted data and assessed the risk of bias in duplicate and independently and updated the evidence reports accordingly.

We adopted the source guideline's ratings for the importance of outcomes (e.g., disease activity as a critical outcome; and physical function, radiographic progression, quality of life, and adverse events as important outcomes). For patient values and preferences, we relied on the systematic review on the topic used by the source guidelines [13] and the input of a Saudi patient representative.

The panel relied on cost data collected from the National Unified Procurement Company (NUPCO) for the governmental sector [14] and the Saudi Food Drug Authority (SFDA) for the private sector [15]. For the remaining contextual factors, the panel used their personal knowledge and experience.

### Assessment of the certainty of the body of evidence and strength of recommendation

In the event of no update of the evidence report, we adopted the source guideline's assessments about the certainty of the evidence. For the updated evidence reports, the methodologist members of the coordination group (SY, JK, and EAA) assessed the certainty of the evidence according to the GRADE methodology and using the GRADEpro GDT software (www.gradepro.org) [16]. In addition, and similarly to the source guidelines, we used the GRADE methodology to grade the strength of recommendations [17, 18].

### Decision-making processes

For each question, the coordination group developed GRADE EtD tables using the GRADEpro GDT software (www.gradepro.org), which included both information on health effects from the evidence reports and contextual information. Then, in preparation for the panel meetings, we used the PANELVoice function in GRADEpro GDT to invite panel members to review the EtDs, comment on the included information, and vote on the EtD criteria and recommendations.

During the panel meetings, we used the EtD in GRADEpro as a platform to facilitate the decision-making process. The panel reviewed the pre-meeting votes, discussed, and voted on each of the EtD criteria, then voted on the direction and strength of the recommendations. We used the pre-meeting votes as a starting point for discussions and aimed to reach the final decision by consensus. Although the panel members were aware of the original recommendations, we did not formally consider them when finalizing the adapted recommendations.

### Declaration and management of interests

All panelists were required to declare their interests as relevant to each recommendation. The coordination group developed a form based on the International Committee for Medical Journal Editors (ICMJE) disclosure of interests form [19] and sent it to panel members via email. During the panel meetings, disclosures were shared with the entire panel for every recommendation. In order to minimize the effect of interests, the co-chairs asked the panelists to apply the rule of 'no strong advocacy,' i.e., sharing opinions while avoiding repetition or being verbally forceful (see Additional file 1 for COI declarations).

## Results

**Fig. 1:**
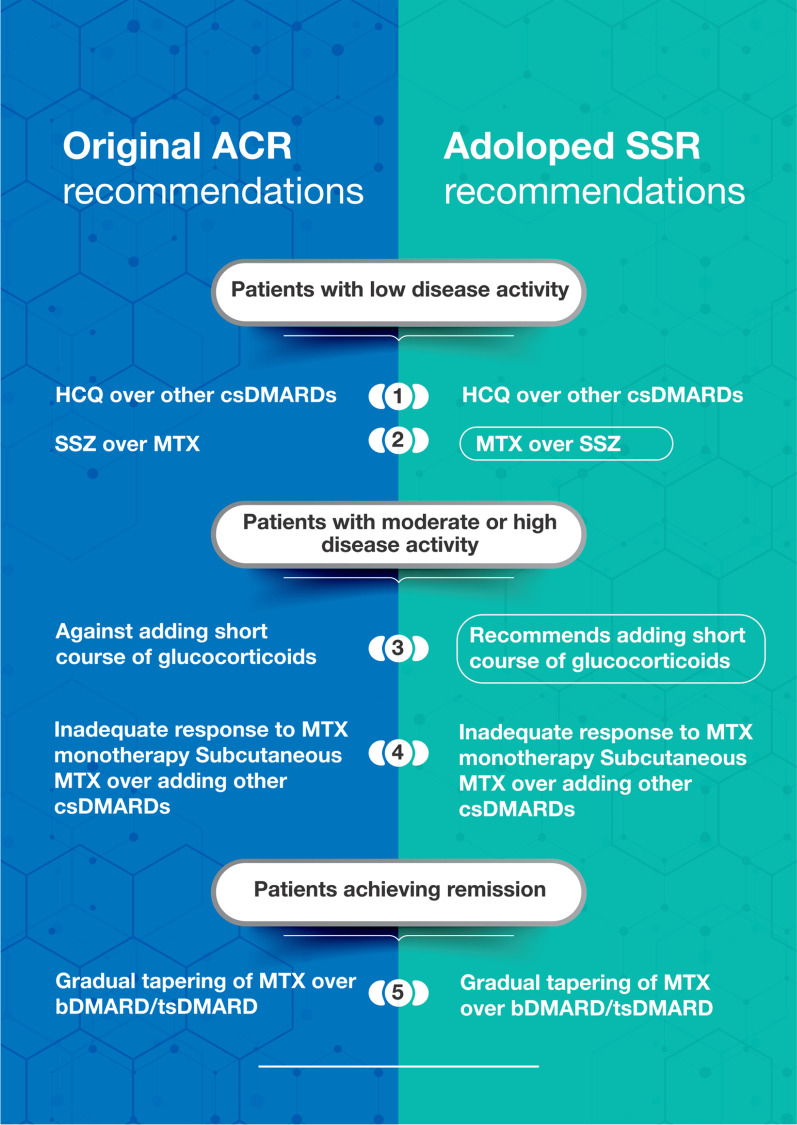
Adoloped SSR recommendations compared to the original ACR recommendations. All recommendations are conditional. The ones circled had their direction changed by the adolopment process. ACR, American College of Rheumatology; SSR, Saudi Society for Rheumatology; HCQ, hydroxychloroquine; SSZ, sulfasalazine; MTX, methotrexate; csDMARDs, conventional synthetic disease modifying anti-rheumatic drugs; bDMARDs, biological DMARDs; tsDMARDs, targeted synthetic DMARDs

Below are the five adapted recommendations, associated remarks, and justifications. Figure 1 presents these recommendations compared to the original recommendations. Additional file 3 includes the EtDs for each recommendation. The panel adopted recommendations from the 2021 ACR guidelines not prioritized for adolopment as part of this project.

### Recommendation 1

The Saudi panel suggests using methotrexate (MTX) over sulfasalazine (SSZ) in DMARD-naive patients with low disease activity (conditional recommendation; based on very low certainty evidence).

#### Remarks

- Recommendation modified (from conditional in favor of SSZ to conditional in favor of MTX); certainty of evidence unmodified.
- This recommendation applies to patients with low disease activity for which medication treatment is judged to be necessary.
- The choice should account for the patient's views on the expected benefits and harms of the respective medications.
- The choice should consider the availability of the medications.
- It is important to monitor adverse events and adjust medications accordingly.

Rationale: The panel favored MTX over SSZ because of the dosing convenience of MTX (once weekly) and its lower cost compared with SSZ.

### Recommendation 2

The Saudi panel suggests using hydroxychloroquine (HCQ) over SSZ in DMARD-naive patients with low disease activity (conditional recommendation; based on very low certainty evidence).

#### Remarks

- Recommendation unmodified; certainty of evidence unmodified.
- This recommendation applies to patients with low disease activity for which medication treatment is judged to be necessary.
- The choice should account for the patient's views on the expected benefits and harms of the respective medications.
- The choice should consider the availability of the medications.
- It is important to monitor adverse events and adjust medications accordingly.

Rationale: The panel favored HCQ over SSZ because of the dosing convenience (once daily) and the safety profile of HCQ compared to SSZ.

### Recommendation 3

The Saudi panel suggests initiating a csDMARD with short-term (< 3 months) glucocorticoids over initiating a csDMARD without short-term glucocorticoids in DMARD-naive patients with moderate-to-high disease activity (conditional recommendation, based on very low certainty evidence).

#### Remarks

- Recommendation modified (from conditional against glucocorticoids to conditional in favor of glucocorticoids); certainty of evidence unmodified.
- The physician should clearly communicate to the patient the potential benefits and harms of glucocorticoids.
- The choice should account for the patient's views on the expected benefits and harms of glucocorticoids. It should also account for the ability to taper/discontinue glucocorticoids.
- The glucocorticoid treatment regimen should use the lowest dose for the shortest period possible to reduce harm, with caution in the elderly.
- It is important to monitor for the adverse events of glucocorticoids.

Rationale: The use of short-term (< 3 months) glucocorticoids when initiating a csDMARD was judged by the panel to be the standard of care. The panel judged the benefit of rapid alleviation of symptoms at the time of diagnosis and during flares to outweigh the risks of glucocorticoids. The panel unanimously agreed that the long-term use of glucocorticoids is associated with harm and damage that outweigh benefit. The panel recommended a shared decision making approach in decisions related to initiating and discontinuing glucocorticoids.

### Recommendation 4

The Saudi panel suggests a switch to subcutaneous MTX over addition/switch to alternative DMARD(s) in patients taking oral MTX who are not at target (conditional recommendation, based on very low certainty evidence).

#### Remarks

- Recommendation unmodified; certainty of evidence unmodified.
- This recommendation typically applies to patients with moderate or high disease activity but may apply to patients with low disease activity.
- Target may differ based on initial disease activity status.
- The choice should consider the availability of the medications.
- It is important to monitor adverse events and adjust the regimen accordingly.

Rationale: The panel favored switching to subcutaneous MTX over addition/switch to alternative DMARD(s) because of the convenience of once-weekly dosing of subcutaneous MTX compared to daily dosing of alternative DMARD(s), and the higher effectiveness of subcutaneous MTX.

### Recommendation 5

The Saudi panel suggests gradual discontinuation of MTX over gradual discontinuation of the bDMARD or tsDMARD for patients taking MTX plus a bDMARD or tsDMARD who wish to discontinue a DMARD (conditional recommendation, based on moderate certainty evidence).

#### Remarks

- Recommendation unmodified; certainty of evidence modified (from very low to moderate).
- Patient’s health coverage in the private sector should be considered when applying this recommendation.
- The patient and the physician should closely monitor the progression of symptoms during and following discontinuation.

Rationale: The recommendation was based on newly identified evidence suggesting decreased disease worsening with gradual discontinuation of MTX compared to gradual discontinuation of the bDMARD or tsDMARD.

## Discussion

### Summary

We describe the adaptation of five prioritized recommendations from the 2021 ACR guideline for the treatment of RA for the context of Saudi Arabia, following GRADE-ADOLOPMENT methodology. For each recommendation, an EtD table was produced using evidence as published in the source guideline, newly published relevant studies solicited by the panel, and contextual information from Saudi Arabia.

### Modifications to the recommendations

Out of five adoloped recommendations, the panel modified two (#1 and #3). For recommendation 1, the panel judged that a generally low rate of adherence to medications in Saudi Arabia favored MTX (administered weekly) over SSZ (administered twice daily). In addition, the cost of a 12-week treatment course of SSZ is substantially higher than that of MTX. Moreover, the KSA panel judged that alcohol-related undesirable effects of MTX were less relevant to the KSA setting given restrictions on alcohol use. These factors tilted the balance of desirable and undesirable effects in favor of MTX.

As for recommendation 3, the KSA panel judged that the balance of effects “probably favors” short-term (< 3 months) glucocorticoids when initiating a csDMARD. Although valuation information by the ACR panel for this specific recommendation is not available, the KSA panel highly valued rapid alleviation of symptoms at the time of diagnosis and during flares. Still, the KSA panel acknowledged challenges with glucocorticoid treatment and detailed them in the rationale as a note of caution to users.

An additional contextual factor considered by the KSA panel was the increased risk of hemolysis with the use of HCQ in Saudi Arabia due to the relatively high prevalence of glucose-6-phosphatedehydrogenase (G6PD) deficiency in this population [20]. However, it did not lead to a modification of recommendation 2, as the panel still had higher safety concerns for SSZ.

### Implementation

The implementation of these five adoloped recommendations should help with standardizing the care of patients with RA in Saudi Arabia. In fact, these recommendations were based not only on a synthesis of the best available evidence, but also on a careful consideration of the Saudi Arabian context. The SSR will lead efforts to disseminate these five adoloped recommendations, as well as the remaining recommendations adopted from the 2021 ACR guidelines.

There are two important factors that would enhance the implementation of the recommendations. First, the specific management choice should account for the patient's views on the expected benefits and harms of the respective alternatives. Second, the choice should consider the availability of the medications, given the variability across regions and health systems. Indeed, in a national survey of Saudi rheumatologists conducted in 2014, 24% of respondents reported not using parenteral MTX because of unavailability in the hospital formulary [21]. It is believed that this percentage has decreased since the introduction of MTX into the MOH formulary.

For recommendation 5, the decision of tapering csDMARDs is associated with an increased cost in the short term compared to tapering bDMARDs or tsDMARDs. In patients who pay from pocket or have a co-pay insurance system, tapering bDMARDs/tsDMARDs would be a reasonable option. Of note, non-profit organizations such as the Charitable Association for Rheumatic Diseases provide free-of-charge biologics that can help clinicians provide the best standard of care regarding drug tapering.

Previously, members of this group contributed to an adolopment exercise of the 2015 ACR RA guidelines for the Middle East and North Africa region [22, 23]. Unlike this project, the previous one was not led or adopted by a medical professional society. We hope that the leading role of SSR on the current project will enhance its dissemination and implementation of the recommendations.

### Suggestions for further research

There is a need for research on how the target population values the outcomes of interest. It is also important to define 'decision thresholds' for judging the extent of benefits and harms (e.g., trivial, small, moderate, or large) based on the reported effect measures (e.g., absolute effects for dichotomous outcomes). The SSR will aim to update these recommendations in parallel to future updates by the ACR. Future efforts should address questions related to the use of biosimilar [24, 25], an important issue to rheumatology practice in the KSA not covered in the source guidelines.

## Conclusion

Rheumatologists practicing in Saudi Arabia can use the five adoloped recommendations generated by this project while adopting the rest of the recommendations from the 2021 ACR guidelines.

## Supplementary Information

**Additional file 1.** List of KSA panel members.

**Additional file 2.** Prioritized questions for the adolopment of the 2021 American College of Rheumatology (ACR) Guideline for the Treatment of Rheumatoid Arthritis (RA) in Saudi Arabia.

**Additional file 3.** Evidence to Decision tables.

## Data Availability

Not applicable (no new data were created or analyzed).

## Abbreviations

ACR: American College of Rheumatology
bDMARD: Biologic Disease-Modifying Anti-Rheumatic Drug
csDMARD: Conventional synthetic Disease-Modifying Anti-Rheumatic Drug
EULAR: European League Against Rheumatism
EtD: Evidence to decision
G6PD: Glucose-6-Phosphate Dehydrogenase
GRADE: Grading of Recommendations Assessment, Development and Evaluation
HCQ: Hydroxychloroquine
KSA: Kingdom of Saudi Arabia
MTX: Methotrexate
RA: Rheumatoid arthritis
SSR: Saudi Society for Rheumatology
SSZ: Sulfasalazine
tsDMARD: Targeted synthetic Disease-Modifying Anti-Rheumatic Drugs
