# Supplementary material for: Recommendations for the treatment of rheumatoid arthritis in Saudi Arabia: adolopment of the 2021 American College of Rheumatology guidelines
Source: BMC Rheumatol. 2022 Nov 23;6:70. doi: 10.1186/s41927-022-00301-y (PMC9682746; doi:10.1186/s41927-022-00301-y)
Supplement: Supplementary file 1 — Additional file 1. List of KSA panel members. [file 41927_2022_301_MOESM1_ESM.docx]

**Appendix 1:** List of KSA panel members

| **Name and title** | **Affiliation(s)** | **Stakeholder group** | **Conflicts of interest** |
| --- | --- | --- | --- |
| Sultana Abdulaziz, MBBS, ABIM, JBIM, RF-KFSH&RC | Division of Rheumatology, Department of Medicine, King Fahad Hospital, Jeddah, Saudi Arabia | Rheumatologist | None |
| Ghada A. Al Janobi, MBBS, SBIM, SF Rheum | Rheumatology Unit,  Department of Medicine, Qatif Central Hospital, Qatif, Saudi Arabia | Rheumatologist | None |
| Abdulaziz Al Khalaf, MBBS, SF Rheum | Rheumatology Unit, Department of Medicine, King Saud University, Riyadh, Saudi Arabia | Rheumatologist | Relevant to switch to SC MTX vs addition/switch to alternative DMARD(s):   - Payment or honoraria for lectures, presentations, speakers bureaus, manuscript writing or educational events (Pfizer, Abbvie, Lilly)   Relevant to gradual discontinuation of MTX vs bDMARD/tsDMARD:   - Grants or contracts (Pfizer, Lilly, Abbvie) - Support for attending meetings and/or travel (Pfizer, Abbvie, Lilly) - Participation on a Data Safety Monitoring Board or Advisory Board (Lilly, Pfizer) - Fellowship training program support (Pfizer) |
| Bader Al Mehmadi, MBBS, SBIM, SF Rheum | Division of Rheumatology, Department of Medicine, College of Medicine, Majmaah University, Al-Majmaah, 11952, Saudi Arabia | Rheumatologist | None |
| Mahasin Al Nassar, MBBS | Department of Obstetrics and Gynecology, King Saud University, Riyadh, Saudi Arabia | Patient representative | None |
| Hanan Al Rayes, MD, MBBS,  ABIM  SF Rheum | Division of Rheumatology, Department of Internal Medicine, Prince Sultan Military Medical City, Riyadh, Saudi Arabia | Rheumatologist | Not relevant to any of the recommendations:   - Honoraria for lectures and speaker fees (Pfizer, AbbVie, Lilly and Janssen) - Consulting fees (Pfizer, Lilly, AbbVie, Amgen, and Janssen) |
| Faisal AlBalawi, MBBS, SBIM, SF Rheum | Section of Rheumatology, Department of Internal Medicine , King Fahad Medical City-Riyadh, Saudi Arabia | Rheumatologist | None |
| Abdullah S. AlFurayj, MBBS, SBIM, SF Rheum | Rheumatology Unit, Department of Medicine, Buraidah Central Hospital, B.C.H, Qassim, Buraidah, Saudi Arabia | Rheumatologist | None |
| Ahmed Hamdan Al-Jedai, Pharm.D., M.B.A., BCPS, FCCP, FAST, FASHP | Deputyship of Therapeutic Affairs, Ministry of Health, Riyadh, Saudi Arabia; College of Medicine, Alfaisal University, Riyadh, Saudi Arabia | Policymaker | None |
| Haya Mohammed Almalag, MSc, PhD | Department of Clinical Pharmacy, College of Pharmacy, King Saud University , Riyadh, Saudi Arabia | Pharmacist | None |
| Hajer Yousef Almudaiheem, Pharm.D., MSc. | Deputyship of Therapeutic affairs, Ministry of Health, Riyadh, Saudi Arabia | Policymaker | None |
| Ali  AlRehaily, M.D, FRCPC,  MMedEdu | Department of Medicine, Section of Rheumatology, Security Forces Hospital Program, Riyadh, Saudi Arabia | Rheumatologist | None |
| Mohammed A. Attar, MBBS, SBIM | Division of Rheumatology, Department of Medicine, Al Hada Armed Forces Hospital, Taif, Saudi Arabia. | Rheumatologist | Relevant to gradual discontinuation of MTX vs bDMARD/tsDMARD:  Support for attending meetings and/or travel: Abbvie (Open Rheum meeting, Jeddah), Lilly (ADARCC, Dubai) |
| Lina El Kibbi, MBBS | Division of Rheumatology, Department of Internal Medicine, Specialized Medical Center, Riyadh, Saudi Arabia | Rheumatologist | None |
| Liana Fraenkel, MD, MPH | Berkshire Medical Center, Pittsfield, Massachusetts, and Yale University School of Medicine, New Haven, Connecticut, United States | Rheumatologist | None |
| Hussein Halabi, MBBS | Section of Rheumatology, Department of Internal Medicine, King Faisal Specialist Hospital and Research Center-Jeddah, Saudi Arabia | Rheumatologist | None |
| Manal Hasan, MBBS | Division of Rheumatology, Department of Internal Medicine, Imam Abdulrahman Bin Faisal University, Dammam, Saudi Arabia | Rheumatologist | None |
| Mohammed A. Omair, MBBS, SF Rheum | Rheumatology Unit, Department of Medicine, King Saud University, Riyadh, Saudi Arabia | Rheumatologist | Relevant to gradual discontinuation of MTX vs bDMARD/tsDMARD:   - Grants or contracts (Abbvie, Pfizer, New Bridge, Roche and BMS) - Consulting fees (Abbvie, Actelion, Amgen, Bristol Myers Squibb, GSK, Hekma, Hoffman- La Roche, New Bridge, Pfizer) - Honoraria for lectures, presentations, speakers bureaus, manuscript writing or educational events (Abbvie, Actelion, Amgen, Bristol Myers Squibb, GSK, Hekma, Hoffman- La Roche, New Bridge, Pfizer - Support for attending meetings and/or travel (Abbvie, Actelion, Amgen, Bristol Myers Squibb, Gsk, Hekma, Hoffman- La Roche, New Bridge, Pfizer) |
| Jasvinder A. Singh, MBBS, MPH | Medicine Service, VA Medical Center, 700 19th St S, Birmingham, AL 35233 USA; Department of Medicine at the School of Medicine, University of Alabama at Birmingham (UAB), 510 20th Street S, Birmingham, AL 35294-0022, USA; Department of Epidemiology at the UAB School of Public Health, 1665 University Blvd., Ryals Public Health Building, Birmingham, AL 35294-0022, USA. | Rheumatologist | Not relevant to any of the recommendations:   - Consulting fees: Crealta/Horizon, Medisys, Fidia, PK Med, Two labs Inc., Adept Field Solutions, Clinical Care options, Clearview healthcare partners, Putnam associates, Focus forward, Navigant consulting, Spherix, MedIQ, Jupiter Life Science, UBM LLC, Trio Health, Medscape, WebMD, and Practice Point communications; and the National Institutes of Health and the American College of Rheumatology - Payment or honoraria for lectures, presentations, speakers bureaus, manuscript writing or educational events: on the speaker’s bureau of Simply Speaking - Support for attending meetings and/or travel: support from OMERACT, an international organization that develops measures for clinical trials and receives arm’s length funding from 12 pharmaceutical companies, to attend their meeting every 2 years - Leadership or fiduciary role in other board, society, committee or advocacy group, paid or unpaid: steering committee member of the OMERACT, an international organization that develops measures for clinical trials and receives arm’s length funding from 12 pharmaceutical companies; chair of the Veterans Affairs Rheumatology Field Advisory Committee; editor and the Director of the UAB Cochrane Musculoskeletal Group Satellite Center on Network Meta-analysis - Stock or stock options: currently owned stock in TPT Global Tech, Vaxart pharmaceuticals, Atyu biopharma, Adaptimmune Therapeutics, GeoVax Labs, Pieris Pharmaceuticals, Enzolytics Inc., Seres Therapeutics and Charlotte’s Web Holdings, Inc; previously owned stock options in Amarin, Viking and Moderna pharmaceuticals |
