# Supplementary material for: Recommendations for the treatment of rheumatoid arthritis in Saudi Arabia: adolopment of the 2021 American College of Rheumatology guidelines
Source: BMC Rheumatol. 2022 Nov 23;6:70. doi: 10.1186/s41927-022-00301-y (PMC9682746; doi:10.1186/s41927-022-00301-y)
Supplement: Supplementary file 2 — Additional file 2. Prioritized questions for the adolopment of the 2021 American College of Rheumatology (ACR) Guideline for the Treatment of Rheumatoid Arthritis (RA) in Saudi Arabia. [file 41927_2022_301_MOESM2_ESM.docx]

**Appendix 2:** Prioritized questions for the adolopment of the 2021 American College of Rheumatology (ACR) Guideline for the Treatment of Rheumatoid Arthritis (RA) in Saudi Arabia

**Prioritized question #1:**

In DMARD-naive patients with low disease activity, should treatment be initiated with methotrexate versus sulfasalazine?

| **P**opulation | DMARD-naive patients with low disease activity |
| --- | --- |
| **I**ntervention | Initiate treatment with methotrexate |
| **C**omparator | Initiate treatment with sulfasalazine |

This question was derived from the following ACR recommendation:

Hydroxychloroquine is conditionally recommended over other csDMARDs, sulfasalazine is conditionally recommended over methotrexate, and methotrexate is conditionally recommended over leflunomide for DMARD-naïve patients with low disease activity

**Prioritized question #2:**

In DMARD-naive patients with low disease activity, should treatment be initiated with hydroxychloroquine versus sulfasalazine?

| **P**opulation | DMARD-naive patients with low disease activity |
| --- | --- |
| **I**ntervention | Initiate treatment with hydroxychloroquine |
| **C**omparator | Initiate treatment with sulfasalazine |

This question was derived from the following ACR recommendation:

Hydroxychloroquine is conditionally recommended over other csDMARDs, sulfasalazine is conditionally recommended over methotrexate, and methotrexate is conditionally recommended over leflunomide for DMARD-naïve patients with low disease activity

**Prioritized question #3:**

In DMARD-naïve patients with moderate-to-high disease activity, should treatment with csDMARDs be initiated with short-term (< 3 months) glucocorticoids versus without short-term (< 3 months) glucocorticoids?

| **P**opulation | DMARD-naïve patients with moderate-to-high disease activity |
| --- | --- |
| **I**ntervention | Initiation of a csDMARD with short-term (< 3 months) glucocorticoids |
| **C**omparator | Initiation of a csDMARD without short-term (< 3 months) glucocorticoids |

This question was derived from the following ACR recommendation:

Initiation of a csDMARD without short-term (<3 months) glucocorticoids is conditionally recommended over initiation of a csDMARD with short-term glucocorticoids for DMARD-naïve patients with moderate-to-high disease activity.

**Prioritized question #4:**

Should patients on oral methotrexate who are not at target switch to subcutaneous methotrexate versus add/switch to alternative DMARD(s)?

| **P**opulation | Patients on oral methotrexate who are not at target |
| --- | --- |
| **I**ntervention | Switch to subcutaneous methotrexate |
| **C**omparator | Add/switch to alternative DMARD(s) |

This question was derived from the following ACR recommendation:

For patients taking oral methotrexate who are not at target, a switch to subcutaneous methotrexate is conditionally recommended over addition/switch to alternative DMARD(s).

**Prioritized question #5:**

Should patients taking methotrexate plus a bDMARD or tsDMARD who are at target taper off methotrexate or taper off the bDMARD or the tsDMARD?

| **P**opulation | Patients taking methotrexate plus a bDMARD or methotrexate plus a tsDMARD who are at target |
| --- | --- |
| **I**ntervention | Gradual discontinuation of methotrexate |
| **C**omparator | Gradual discontinuation of the bDMARD or tsDMARD |

This question was derived from the following ACR recommendation:

For patients taking methotrexate plus a bDMARD or tsDMARD who wish to discontinue a DMARD, gradual discontinuation of methotrexate is conditionally recommended over gradual discontinuation of the bDMARD or tsDMARD.

Outcomes considered for all above questions are:

1. Disease activity
2. Function or functional ability
3. Preventing joint damage
4. Serious adverse events
5. Reversible side effects that are not “serious” but that impact QOL
6. Percent stopping medication because of side effects
7. Quality of life
