# Supplementary material for: Recommendations for the treatment of rheumatoid arthritis in Saudi Arabia: adolopment of the 2021 American College of Rheumatology guidelines
Source: BMC Rheumatol. 2022 Nov 23;6:70. doi: 10.1186/s41927-022-00301-y (PMC9682746; doi:10.1186/s41927-022-00301-y)
Supplement: Supplementary file 3 — Additional file 3. Evidence to Decision tables. [file 41927_2022_301_MOESM3_ESM.docx]

**Appendix 3:** Evidence to Decision tables

| QUESTION | |
| --- | --- |
| **Should methotrexate monotherapy vs. sulfasalazine be used for DMARD-naive patients with low disease activity?** | |
| **POPULATION:** | DMARD-naive patients with low disease activity |
| **INTERVENTION:** | methotrexate monotherapy |
| **COMPARISON:** | sulfasalazine |
| **MAIN OUTCOMES:** | Disease activity (follow up: 1 year; assessed with: DAS28-ESR (Lower values –> benefit) (MCID -1.17); Disability (follow up: 1 year; assessed with: HAQ-DI (Lower values –> benefit) (MCID -0.22); Pain (follow up: 1 year; assessed with: VAS 0-100 (Lower values – > benefit) (MCID -11.9); Serious adverse events (follow up: 1 year); Withdrawal due to lack of efficacy (follow up: 1 year); Withdrawal due to adverse events (follow up: 1 year); |
| **SETTING:** |  |
| **PERSPECTIVE:** |  |
| **BACKGROUND:** |  |
| **CONFLICT OF INTERESTS:** |  |

# ASSESSMENT

| Desirable Effects How substantial are the desirable anticipated effects? | | |
| --- | --- | --- |
| JUDGEMENT | RESEARCH EVIDENCE | ADDITIONAL CONSIDERATIONS |
| ○ Trivial ● Small ○ Moderate ○ Large ○ Varies ○ Don't know | \| **Outcomes** \| **№ of participants (studies) Follow-up** \| **Certainty of the evidence (GRADE)** \| **Relative effect (95% CI)** \| **Anticipated absolute effects^*^ (95% CI)** \| \| \| --- \| --- \| --- \| --- \| --- \| --- \| \| **Risk with sulfasalazine** \| **Risk difference with methotrexate monotherapy** \| \| Disease activity (follow up: 1 year; assessed with: DAS28-ESR (Lower values –> benefit) (MCID -1.17) \| 206 (2 RCTs) \| ⨁◯◯◯ Very low^a,b,c,d^ \| - \| The mean disease activity (follow up: 1 year; assessed with: DAS28-ESR (Lower values –> benefit) (MCID -1.17) was **0** \| MD **0.14 higher** (0.18 lower to 0.47 higher) \| \| Disability (follow up: 1 year; assessed with: HAQ-DI (Lower values –> benefit) (MCID -0.22) \| 206 (2 RCTs) \| ⨁◯◯◯ Very low^a,c,d^ \| - \| The mean disability (follow up: 1 year; assessed with: HAQ-DI (Lower values –> benefit) (MCID -0.22) was **0** \| MD **0.04 fewer** (0.2 fewer to 0.13 more) \| \| Pain (follow up: 1 year; assessed with: VAS 0-100 (Lower values – > benefit) (MCID -11.9) \| 69 (1 RCT) \| ⨁◯◯◯ Very low^c,d^ \| - \| The mean pain (follow up: 1 year; assessed with: VAS 0-100 (Lower values – > benefit) (MCID -11.9) was **0** \| MD **0.1 higher** (13.46 lower to 13.66 higher) \| \| Withdrawal due to lack of efficacy (follow up: 1 year) \| 206 (2 RCTs) \| ⨁◯◯◯ Very low^a,c,e^ \| **RR 0.51** (0.19 to 1.39) \| Study population \| \| \| 98 per 1,000 \| **48 fewer per 1,000** (79 fewer to 38 more) \|   **Explanations**   1. Downgraded by one level due to serious risk of bias. Lack of allocation and lack of blinding of outcome assessors of non-radiographic outcomes in the study with the higher weight. 2. I2= 41% 3. Downgraded by one level due to serious indirectness. The evidence is based on a population with moderate to high disease activity. 4. Downgraded by one level due to serious imprecision. Small sample size. 5. Downgraded by two levels due to very serious imprecision. Confidence interval includes both values suggesting benefit and values suggesting harm. Small sample size and low number of events. | - Dose of MTX used in trials is lower than the one used in practice. - Disease activity is not generally measured by clinicians in the KSA context. - Rate of adherence to medications is generally low. - Disease activity due to inflammation is considered regardless of disease duration.     Trivial: 1/17 (6%)  Small: 11/17 (65%)  Moderate: 4/17 (24%)  Large: 1/17 (6%)  Varies: 0/17 (0%)  Don't know: 0/17 (0%) |
| Undesirable Effects How substantial are the undesirable anticipated effects? | | |
| JUDGEMENT | RESEARCH EVIDENCE | ADDITIONAL CONSIDERATIONS |
| ○ Large ○ Moderate ○ Small ● Trivial ○ Varies ○ Don't know | \| **Outcomes** \| **№ of participants (studies) Follow-up** \| **Certainty of the evidence (GRADE)** \| **Relative effect (95% CI)** \| **Anticipated absolute effects^*^ (95% CI)** \| \| \| --- \| --- \| --- \| --- \| --- \| --- \| \| **Risk with sulfasalazine** \| **Risk difference with methotrexate monotherapy** \| \| Serious adverse events (follow up: 1 year) \| 69 (1 RCT) \| ⨁◯◯◯ Very low^a,b^ \| **RR 0.14** (0.01 to 2.59) \| Study population \| \| \| 88 per 1,000 \| **76 fewer per 1,000** (87 fewer to 140 more) \| \| Withdrawal due to adverse events (follow up: 1 year) \| 206 (2 RCTs) \| ⨁◯◯◯ Very low^a,c,d,e^ \| **RR 0.46** (0.22 to 0.98) \| Study population \| \| \| 186 per 1,000 \| **101 fewer per 1,000** (145 fewer to 4 fewer) \|   **Explanations**   1. Downgraded by one level due to serious indirectness. The evidence is based on a population with moderate to high disease activity. 2. Downgraded by two levels due to very serious imprecision. Confidence interval includes both values suggesting benefit and values suggesting harm. Very small sample size. 3. Downgraded by one level due to serious risk of bias. Lack of allocation and lack of blinding of outcome assessors of non-radiographic outcomes in the study with the higher weight. 4. I2= 44% 5. Downgraded by one level due to serious imprecision. Confidence interval includes both values suggesting benefit and values suggesting no effect. Small sample size. | - Alcohol-related adverse events of MTX might be less important due to restrictions on alcohol use   Large: 0/16 (0%)  Moderate: 0/16 (0%)  Small: 6/16 (38%)  Trivial: 10/16 (63%)  Varies: 0/16 (0%)  Don't know: 0/16 (0%) |
| Certainty of evidence What is the overall certainty of the evidence of effects? | | |
| JUDGEMENT | RESEARCH EVIDENCE | ADDITIONAL CONSIDERATIONS |
| ● Very low ○ Low ○ Moderate ○ High ○ No included studies |  |  |
| Values Is there important uncertainty about or variability in how much people value the main outcomes? | | |
| JUDGEMENT | RESEARCH EVIDENCE | ADDITIONAL CONSIDERATIONS |
| ○ Important uncertainty or variability ● Possibly important uncertainty or variability ○ Probably no important uncertainty or variability ○ No important uncertainty or variability | • Patient preferences are variable  • Treatment benefit is more important than non-serious and serious adverse events  • Treatment benefit is more important than route of administration  • Route of administration is often more important than serious/non-serious adverse events | - Essential for clinicians to engage patients in clarifying their values.   Important uncertainty or variability: 2/16 (13%)  Possibly important uncertainty or variability: 11/16 (69%)  Probably no important uncertainty or variability: 3/16 (19%)  No important uncertainty or variability: 0/16 (0%) |
| Balance of effects Does the balance between desirable and undesirable effects favor the intervention or the comparison? | | |
| JUDGEMENT | RESEARCH EVIDENCE | ADDITIONAL CONSIDERATIONS |
| ○ Favors the comparison ○ Probably favors the comparison ○ Does not favor either the intervention or the comparison ● Probably favors the intervention ○ Favors the intervention ○ Varies ○ Don't know |  | Favors the comparison: 1/16 (6%)  Probably favors the comparison: 0/16 (0%)  Does not favor either the intervention or the comparison: 3/16 (19%)  Probably favors the intervention: 10/16 (63%)  Favors the intervention: 2/16 (13%)  Varies: 0/16 (0%)  Don't know: 0/16 (0%) |
| Resources required How large are the resource requirements (costs)? | | |
| JUDGEMENT | RESEARCH EVIDENCE | ADDITIONAL CONSIDERATIONS |
| ○ Large costs ○ Moderate costs ○ Negligible costs and savings ● Moderate savings ○ Large savings ○ Varies ○ Don't know | 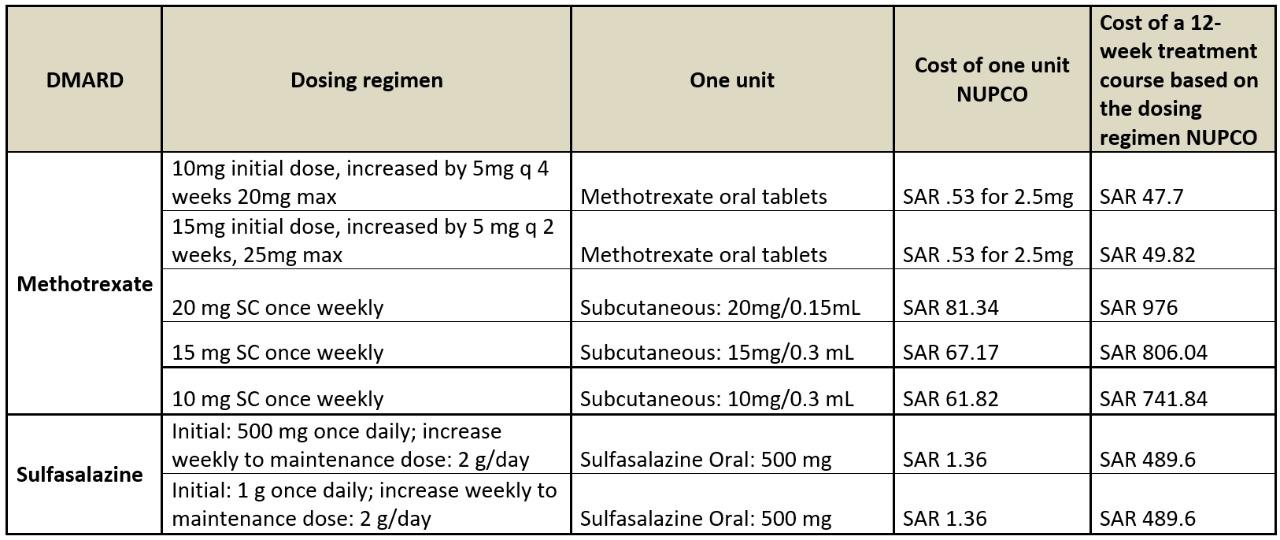 | - Judgmennts considered oral dosing. - Many regions or healthcare systems (e.g. MOH) have no access to SC. - Saving with oral; cost with SC - There might be scenarios where it is needed to switch from oral to SC (e.g., absorption problem)   Large costs: 0/16 (0%)  Moderate costs: 1/16 (6%)  Negligible costs and savings 1/16 (6%)  Moderate savings: 7/16 (44%)  Large savings: 7/16 (44%)  Varies: 0/16 (0%)  Don't know: 0/16 (0%) |
| Cost effectiveness Does the cost-effectiveness of the intervention favor the intervention or the comparison? | | |
| JUDGEMENT | RESEARCH EVIDENCE | ADDITIONAL CONSIDERATIONS |
| ○ Favors the comparison ○ Probably favors the comparison ○ Does not favor either the intervention or the comparison ● Probably favors the intervention ○ Favors the intervention ○ Varies ○ No included studies |  | Favors the comparison: 0/16 (0%)  Probably favors the comparison: 1/16 (6%)  Does not favor either the intervention or the comparison: 1/16 (6%)  Probably favors the intervention: 11/16 (69%)  Favors the intervention: 1/16 (6%)  Varies: 0/16 (0%)  No included studies: 2/16 (13%) |
| Equity What would be the impact on health equity? | | |
| JUDGEMENT | RESEARCH EVIDENCE | ADDITIONAL CONSIDERATIONS |
| ○ Reduced ○ Probably reduced ○ Probably no impact ● Probably increased ○ Increased ○ Varies ○ Don't know |  | Reduced: 0/16 (0%)  Probably reduced: 1/16 (6%)  Probably no impact: 5/16 (31%)  Probably increased: 9/16 (56%)  Increased: 1/16 (6%)  Varies: 0/16 (0%)  Don't know: 0/16 (0%) |
| Acceptability Is the intervention acceptable to key stakeholders? | | |
| JUDGEMENT | RESEARCH EVIDENCE | ADDITIONAL CONSIDERATIONS |
| ○ No ○ Probably no ● Probably yes ○ Yes ○ Varies ○ Don't know |  | - MTX is administered weekly, whereas SSZ is administered twice daily     No: 0/16 (0%)  Probably no: 0/16 (0%)  Probably yes: 9/16 (56%)  Yes: 4/16 (25%)  Varies: 2/16 (13%)  Don't know: 1/16 (6%) |
| Feasibility Is the intervention feasible to implement? | | |
| JUDGEMENT | RESEARCH EVIDENCE | ADDITIONAL CONSIDERATIONS |
| ○ No ○ Probably no ● Probably yes ○ Yes ○ Varies ○ Don't know |  | No: 0/17 (0%)  Probably no: 0/17 (0%)  Probably yes: 9/17 (53%)  Yes: 8/17 (47%)  Varies: 0/17 (0%)  Don't know: 0/17 (0%) |

# SUMMARY OF JUDGEMENTS

|  | **JUDGEMENT** | | | | | | |
| --- | --- | --- | --- | --- | --- | --- | --- |
| **DESIRABLE EFFECTS** | Trivial | **Small** | Moderate | Large |  | Varies | Don't know |
| **UNDESIRABLE EFFECTS** | Large | Moderate | Small | **Trivial** |  | Varies | Don't know |
| **CERTAINTY OF EVIDENCE** | **Very low** | Low | Moderate | High |  |  | No included studies |
| **VALUES** | Important uncertainty or variability | **Possibly important uncertainty or variability** | Probably no important uncertainty or variability | No important uncertainty or variability |  |  |  |
| **BALANCE OF EFFECTS** | Favors the comparison | Probably favors the comparison | Does not favor either the intervention or the comparison | **Probably favors the intervention** | Favors the intervention | Varies | Don't know |
| **RESOURCES REQUIRED** | Large costs | Moderate costs | Negligible costs and savings | **Moderate savings** | Large savings | Varies | Don't know |
| **COST EFFECTIVENESS** | Favors the comparison | Probably favors the comparison | Does not favor either the intervention or the comparison | **Probably favors the intervention** | Favors the intervention | Varies | No included studies |
| **EQUITY** | Reduced | Probably reduced | Probably no impact | **Probably increased** | Increased | Varies | Don't know |
| **ACCEPTABILITY** | No | Probably no | **Probably yes** | Yes |  | Varies | Don't know |
| **FEASIBILITY** | No | Probably no | **Probably yes** | Yes |  | Varies | Don't know |

# TYPE OF RECOMMENDATION

| Strong recommendation against the intervention | Conditional recommendation against the intervention | Conditional recommendation for either the intervention or the comparison | **Conditional recommendation for the intervention** | Strong recommendation for the intervention |
| --- | --- | --- | --- | --- |
| ○ | ○ | ○ | **●** | ○ |

# CONCLUSIONS

| Recommendation |
| --- |
| The KSA panel suggests using methotrexate over using sulfasalazine to initiate treatment in DMARD-naive patients with low disease activity (conditional recommendation; based on very low certainty evidence)  Remarks:   - This recommendation applies to patients with low disease activity for which medication treatment is judged to be necessary - The choice should account for the patient's views on the expected benefits and harms of the respective medications - The choice should consider the availability of the medications - It is important to monitor the medications' side effects and adjust regimen accordingly |
|  |
| Justification |
|  |

| Subgroup considerations |
| --- |
|  |
| Implementation considerations |
|  |

| Monitoring and evaluation |
| --- |
|  |
| Research priorities |
|  |

| QUESTION | |
| --- | --- |
| **Should hydroxychloroquine vs. sulfasalazine be used for DMARD-naive patients with low disease activity?** | |
| **POPULATION:** | DMARD-naive patients with low disease activity |
| **INTERVENTION:** | hydroxychloroquine |
| **COMPARISON:** | sulfasalazine |
| **MAIN OUTCOMES:** | Pain (follow up: 11 months; assessed with: VAS 0-10 (Lower values – > benefit) (MCID 0.5); Withdrawal due to lack of efficacy (follow up: 11 months); Withdrawal due to adverse events (follow up: 11 months); |
| **SETTING:** |  |
| **PERSPECTIVE:** |  |
| **BACKGROUND:** |  |
| **CONFLICT OF INTERESTS:** |  |

# ASSESSMENT

| Desirable Effects How substantial are the desirable anticipated effects? | | |
| --- | --- | --- |
| JUDGEMENT | RESEARCH EVIDENCE | ADDITIONAL CONSIDERATIONS |
| ○ Trivial ● Small ○ Moderate ○ Large ○ Varies ○ Don't know | \| **Outcomes** \| **№ of participants (studies) Follow-up** \| **Certainty of the evidence (GRADE)** \| **Relative effect (95% CI)** \| **Anticipated absolute effects^*^ (95% CI)** \| \| \| --- \| --- \| --- \| --- \| --- \| --- \| \| **Risk with sulfasalazine** \| **Risk difference with hydroxychloroquine** \| \| Pain (follow up: 11 months; assessed with: VAS 0-10 (Lower values – > benefit) (MCID 0.5) \| 57 (1 RCT) \| ⨁◯◯◯ Very low^a,b,c^ \| - \| The mean pain (follow up: 11 months; assessed with: VAS 0-10 (Lower values – > benefit) (MCID 0.5) was **0** \| MD **0.02 higher** (1.32 lower to 1.36 higher) \|   **Explanations**   1. Downgraded by one level due to serious risk of bias. Lack of allocation concealment. 2. Downgraded by one level due to serious indirectness. The evidence is based on a population with moderate to high disease activity. 3. Downgraded by two levels due to very serious imprecision. Confidence interval includes both values suggesting harm and values suggesting benefit. Small sample size. | Trivial: 6/17 (35%)  Small: 9/17 (53%)  Moderate: 2/17 (12%)  Large: 0/17 (0%)  Varies: 0/17 (0%)  Don't know: 0/17 (0%) |
| Undesirable Effects How substantial are the undesirable anticipated effects? | | |
| JUDGEMENT | RESEARCH EVIDENCE | ADDITIONAL CONSIDERATIONS |
| ○ Large ○ Moderate ● Small ○ Trivial ○ Varies ○ Don't know | \| **Outcomes** \| **№ of participants (studies) Follow-up** \| **Certainty of the evidence (GRADE)** \| **Relative effect (95% CI)** \| **Anticipated absolute effects^*^ (95% CI)** \| \| \| --- \| --- \| --- \| --- \| --- \| --- \| \| **Risk with sulfasalazine** \| **Risk difference with hydroxychloroquine** \| \| Withdrawal due to lack of efficacy (follow up: 11 months) \| 57 (1 RCT) \| ⨁◯◯◯ Very low^a,b,c^ \| **RR 2.86** (0.87 to 10.00) \| Study population \| \| \| 107 per 1,000 \| **199 more per 1,000** (14 fewer to 964 more) \| \| Withdrawal due to adverse events (follow up: 11 months) \| 57 (1 RCT) \| ⨁◯◯◯ Very low^a,b,c^ \| **RR 0.24** (0.03 to 2.04) \| Study population \| \| \| 143 per 1,000 \| **109 fewer per 1,000** (139 fewer to 149 more) \|   **Explanations**   1. Downgraded by one level due to serious risk of bias. Lack of allocation concealment. 2. Downgraded by one level due to serious indirectness. The evidence is based on a population with moderate to high disease activity. 3. Downgraded by two levels due to very serious imprecision. Confidence interval includes both values suggesting harm and values suggesting benefit. Small sample size. | - Ophthalmologist referrals for monitoring side effects of HCQ - ACR background: recommendation considered the increased burden of side effects with SSZ   Large: 0/16 (0%)  Moderate: 4/16 (25%)  Small: 11/16 (69%)  Trivial: 1/16 (6%)  Varies: 0/16 (0%)  Don't know: 0/16 (0%) |
| Certainty of evidence What is the overall certainty of the evidence of effects? | | |
| JUDGEMENT | RESEARCH EVIDENCE | ADDITIONAL CONSIDERATIONS |
| ● Very low ○ Low ○ Moderate ○ High ○ No included studies |  |  |
| Values Is there important uncertainty about or variability in how much people value the main outcomes? | | |
| JUDGEMENT | RESEARCH EVIDENCE | ADDITIONAL CONSIDERATIONS |
| ○ Important uncertainty or variability ● Possibly important uncertainty or variability ○ Probably no important uncertainty or variability ○ No important uncertainty or variability | • Patient preferences are variable  • Treatment benefit is more important than non-serious and serious adverse events  • Treatment benefit is more important than route of administration  • Route of administration is often more important than serious/non-serious adverse events | Important uncertainty or variability: 2/16 (13%)  Possibly important uncertainty or variability: 11/16 (69%)  Probably no important uncertainty or variability: 3/16 (19%)  No important uncertainty or variability: 0/16 (0%) |
| Balance of effects Does the balance between desirable and undesirable effects favor the intervention or the comparison? | | |
| JUDGEMENT | RESEARCH EVIDENCE | ADDITIONAL CONSIDERATIONS |
| ○ Favors the comparison ○ Probably favors the comparison ○ Does not favor either the intervention or the comparison ● Probably favors the intervention ○ Favors the intervention ○ Varies ○ Don't know |  | - Observed adverse events reflect early phase of treatment (1 year) and these AEs are expected to increase with time for HCQ - More value given to safety (favoring HCQ) relative to efficacy (favoring SSZ) - G6PD deficiency favors starting with SSZ - More compliance with one daily dose (HCQ)   Favors the comparison: 2/17 (12%)  Probably favors the comparison: 4/17 (24%)  Does not favor either the intervention or the comparison: 11/17 (65%)  Probably favors the intervention: 0/17 (0%)  Favors the intervention: 0/17 (0%)  Varies: 0/17 (0%)  Don't know: 0/17 (0%) |
| Resources required How large are the resource requirements (costs)? | | |
| JUDGEMENT | RESEARCH EVIDENCE | ADDITIONAL CONSIDERATIONS |
| ○ Large costs ○ Moderate costs ○ Negligible costs and savings ● Moderate savings ○ Large savings ○ Varies ○ Don't know | 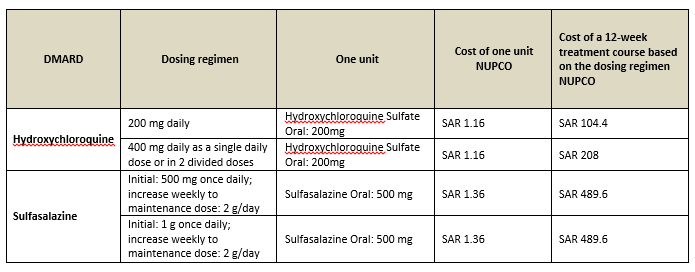 | - NUPCO valid for governement institutions - NUPCO does not apply to the private sector (retail price for Saudi FDA relevant). - SSZ might be cheaper in the private setting - Most expacts are insured through the private sector   Large costs: 0/18 (0%)  Moderate costs: 2/18 (11%)  Negligible costs and savings: 3/18 (17%)  Moderate savings: 11/18 (61%)  Large savings: 0/18 (0%)  Varies: 2/18 (11%)  Don't know: 0/18 (0%) |
| Cost effectiveness Does the cost-effectiveness of the intervention favor the intervention or the comparison? | | |
| JUDGEMENT | RESEARCH EVIDENCE | ADDITIONAL CONSIDERATIONS |
| ○ Favors the comparison ○ Probably favors the comparison ○ Does not favor either the intervention or the comparison ○ Probably favors the intervention ○ Favors the intervention ○ Varies ● No included studies |  | - Keeping in mind the importance of the context (utility valuation and thresholds)   Favors the comparison: 0/19 (0%)  Probably favors the comparison: 1/19 (5%)  Does not favor either the intervention or the comparison: 2/19 (11%)  Probably favors the intervention: 4/19 (21%)  Favors the intervention: 0/19 (0%)  Varies: 0/19 (0%)  No included studies: 12/19 (63%) |
| Equity What would be the impact on health equity? | | |
| JUDGEMENT | RESEARCH EVIDENCE | ADDITIONAL CONSIDERATIONS |
| ○ Reduced ○ Probably reduced ○ Probably no impact ○ Probably increased ○ Increased ● Varies ○ Don't know |  | - Both medications' availablility varies by settings; shortages of HCQ during the pandemic (and some places before the pandemic); SSZ might not be available in specific settings (e.g., in governmental hospitals)   Reduced: 0/19 (0%)  Probably reduced: 0/19 (0%)  Probably no impact: 5/19 (26%)  Probably increased: 6/19 (32%)  Increased: 0/19 (0%)  Varies: 6/19 (32%)  Don't know: 2/19 (11%) |
| Acceptability Is the intervention acceptable to key stakeholders? | | |
| JUDGEMENT | RESEARCH EVIDENCE | ADDITIONAL CONSIDERATIONS |
| ○ No ○ Probably no ● Probably yes ○ Yes ○ Varies ○ Don't know |  | No: 0/19 (0%)  Probably no: 1/19 (5%)  Probably yes: 12/19 (63%)  Yes: 5/19 (26%)  Varies: 0/19 (0%)  Don't know: 1/19 (5%) |
| Feasibility Is the intervention feasible to implement? | | |
| JUDGEMENT | RESEARCH EVIDENCE | ADDITIONAL CONSIDERATIONS |
| ○ No ○ Probably no ● Probably yes ○ Yes ○ Varies ○ Don't know |  | No: 0/19 (0%)  Probably no: 0/19 (0%)  Probably yes: 15/19 (79%)  Yes: 3/19 (16%)  Varies: 1/19 (5%)  Don't know: 0/19 (0%) |

# SUMMARY OF JUDGEMENTS

|  | **JUDGEMENT** | | | | | | |
| --- | --- | --- | --- | --- | --- | --- | --- |
| **DESIRABLE EFFECTS** | Trivial | **Small** | Moderate | Large |  | Varies | Don't know |
| **UNDESIRABLE EFFECTS** | Large | Moderate | **Small** | Trivial |  | Varies | Don't know |
| **CERTAINTY OF EVIDENCE** | **Very low** | Low | Moderate | High |  |  | No included studies |
| **VALUES** | Important uncertainty or variability | **Possibly important uncertainty or variability** | Probably no important uncertainty or variability | No important uncertainty or variability |  |  |  |
| **BALANCE OF EFFECTS** | Favors the comparison | Probably favors the comparison | Does not favor either the intervention or the comparison | **Probably favors the intervention** | Favors the intervention | Varies | Don't know |
| **RESOURCES REQUIRED** | Large costs | Moderate costs | Negligible costs and savings | **Moderate savings** | Large savings | Varies | Don't know |
| **COST EFFECTIVENESS** | Favors the comparison | Probably favors the comparison | Does not favor either the intervention or the comparison | Probably favors the intervention | Favors the intervention | Varies | **No included studies** |
| **EQUITY** | Reduced | Probably reduced | Probably no impact | Probably increased | Increased | **Varies** | Don't know |
| **ACCEPTABILITY** | No | Probably no | **Probably yes** | Yes |  | Varies | Don't know |
| **FEASIBILITY** | No | Probably no | **Probably yes** | Yes |  | Varies | Don't know |

# TYPE OF RECOMMENDATION

| Strong recommendation against the intervention | Conditional recommendation against the intervention | Conditional recommendation for either the intervention or the comparison | **Conditional recommendation for the intervention** | Strong recommendation for the intervention |
| --- | --- | --- | --- | --- |
| ○ | ○ | ○ | **●** | ○ |

# CONCLUSIONS

| Recommendation |
| --- |
| The KSA panel suggests using hydroxychloroquine over using sulfasalazine to initiate treatment in DMARD-naive patients with low disease activity (conditional recommendation; based on very low certainty evidence)  Remarks:   - This recommendation applies to patients with low disease activity for which medication treatment is judged to be necessary - The choice should account for the patient's views on the expected benefits and harms of the respective medications - The choice should consider the availability of the medications - It is important to monitor the medications' side effects and adjust regimen accordingly |
|  |
| Justification |
|  |

| Subgroup considerations |
| --- |
|  |
| Implementation considerations |
|  |

| Monitoring and evaluation |
| --- |
|  |
| Research priorities |
|  |

| QUESTION | |
| --- | --- |
| **Should csDMARD with short-term (<3 months) glucocorticoids vs. csDMARD without short-term (<3 months) glucocorticoids be used for DMARD-naive patients with moderate-to- high disease activity?** | |
| **POPULATION:** | DMARD-naive patients with moderate-to- high disease activity |
| **INTERVENTION:** | csDMARD with short-term (<3 months) glucocorticoids |
| **COMPARISON:** | csDMARD without short-term (<3 months) glucocorticoids |
| **MAIN OUTCOMES:** |  |
| **SETTING:** |  |
| **PERSPECTIVE:** |  |
| **BACKGROUND:** |  |
| **CONFLICT OF INTERESTS:** |  |

# ASSESSMENT

| Desirable Effects How substantial are the desirable anticipated effects? | | |
| --- | --- | --- |
| JUDGEMENT | RESEARCH EVIDENCE | ADDITIONAL CONSIDERATIONS |
| ○ Trivial ○ Small ● Moderate ○ Large ○ Varies ○ Don't know | There are no identified direct (no eligible randomized controlled trials or non-randomized studies) or indirect evidence. | - Pain control   Trivial: 0/17 (0%)  Small: 5/17 (29%)  Moderate: 10/17 (59%)  Large: 0/17 (0%)  Varies: 0/17 (0%)  Don't know: 2/17 (12%) |
| Undesirable Effects How substantial are the undesirable anticipated effects? | | |
| JUDGEMENT | RESEARCH EVIDENCE | ADDITIONAL CONSIDERATIONS |
| ○ Large ○ Moderate ● Small ○ Trivial ○ Varies ○ Don't know | There are no identified direct (no eligible randomized controlled trials or non-randomized studies) or indirect evidence. | - Use the lowest and shortest duration possible to reduce undesirable effects - Particular caution in the elderly   Large: 1/17 (6%)  Moderate: 3/17 (18%)  Small: 13/17 (76%)  Trivial: 0/17 (0%)  Varies: 0/17 (0%)  Don't know: 0/17 (0%) |
| Certainty of evidence What is the overall certainty of the evidence of effects? | | |
| JUDGEMENT | RESEARCH EVIDENCE | ADDITIONAL CONSIDERATIONS |
| ● Very low ○ Low ○ Moderate ○ High ○ No included studies |  |  |
| Values Is there important uncertainty about or variability in how much people value the main outcomes? | | |
| JUDGEMENT | RESEARCH EVIDENCE | ADDITIONAL CONSIDERATIONS |
| ○ Important uncertainty or variability ● Possibly important uncertainty or variability ○ Probably no important uncertainty or variability ○ No important uncertainty or variability | • Patient preferences are variable  • Treatment benefit is more important than non-serious and serious adverse events  • Treatment benefit is more important than route of administration  • Route of administration is often more important than serious/non-serious adverse events | Important uncertainty or variability: 2/16 (13%)  Possibly important uncertainty or variability: 11/16 (69%)  Probably no important uncertainty or variability: 3/16 (19%)  No important uncertainty or variability: 0/16 (0%) |
| Balance of effects Does the balance between desirable and undesirable effects favor the intervention or the comparison? | | |
| JUDGEMENT | RESEARCH EVIDENCE | ADDITIONAL CONSIDERATIONS |
| ○ Favors the comparison ○ Probably favors the comparison ○ Does not favor either the intervention or the comparison ● Probably favors the intervention ○ Favors the intervention ○ Varies ○ Don't know |  | ACR background: The toxicity associated with glucocorticoids was judged to outweigh potential benefits.    Favors the comparison: 0/18 (0%)  Probably favors the comparison: 3/18 (17%)  Does not favor either the intervention or the comparison: 1/18 (6%)  Probably favors the intervention: 13/18 (72%)  Favors the intervention: 1/18 (6%)  Varies: 0/18 (0%)  Don't know: 0/18 (0%) |
| Resources required How large are the resource requirements (costs)? | | |
| JUDGEMENT | RESEARCH EVIDENCE | ADDITIONAL CONSIDERATIONS |
| ○ Large costs ○ Moderate costs ● Negligible costs and savings ○ Moderate savings ○ Large savings ○ Varies ○ Don't know |  | Large costs: 0/17 (0%)  Moderate costs: 0/17 (0%)  Negligible costs and savings: 14/17 (82%)  Moderate savings: 1/17 (6%)  Large savings: 0/17 (0%)  Varies: 2/17 (12%)  Don't know: 0/17 (0%) |
| Cost effectiveness Does the cost-effectiveness of the intervention favor the intervention or the comparison? | | |
| JUDGEMENT | RESEARCH EVIDENCE | ADDITIONAL CONSIDERATIONS |
| ○ Favors the comparison ○ Probably favors the comparison ○ Does not favor either the intervention or the comparison ○ Probably favors the intervention ○ Favors the intervention ○ Varies ● No included studies |  | Favors the comparison: 0/17 (0%)  Probably favors the comparison: 1/17 (6%)  Does not favor either the intervention or the comparison: 1/17 (6%)  Probably favors the intervention: 5/17 (29%)  Favors the intervention: 2/17 (12%)  Varies: 0/17 (0%)  No included studies: 8/17 (47%) |
| Equity What would be the impact on health equity? | | |
| JUDGEMENT | RESEARCH EVIDENCE | ADDITIONAL CONSIDERATIONS |
| ○ Reduced ○ Probably reduced ● Probably no impact ○ Probably increased ○ Increased ○ Varies ○ Don't know |  | Reduced: 0/17 (0%)  Probably reduced: 0/17 (0%)  Probably no impact: 9/17 (53%)  Probably increased: 6/17 (35%)  Increased: 1/17 (6%)  Varies: 0/17 (0%)  Don't know: 1/17 (6%) |
| Acceptability Is the intervention acceptable to key stakeholders? | | |
| JUDGEMENT | RESEARCH EVIDENCE | ADDITIONAL CONSIDERATIONS |
| ○ No ○ Probably no ● Probably yes ○ Yes ○ Varies ○ Don't know |  | - Need for good communication (in terms of side effects)   No: 0/15 (0%)  Probably no: 0/15 (0%)  Probably yes: 12/15 (80%)  Yes: 2/15 (13%)  Varies: 1/15 (7%)  Don't know: 0/15 (0%) |
| Feasibility Is the intervention feasible to implement? | | |
| JUDGEMENT | RESEARCH EVIDENCE | ADDITIONAL CONSIDERATIONS |
| ○ No ○ Probably no ● Probably yes ○ Yes ○ Varies ○ Don't know |  | No: 0/16 (0%)  Probably no: 0/16 (0%)  Probably yes: 10/16 (63%)  Yes: 5/16 (31%)  Varies: 1/16 (6%)  Don't know: 0/16 (0%) |

# SUMMARY OF JUDGEMENTS

|  | **JUDGEMENT** | | | | | | |
| --- | --- | --- | --- | --- | --- | --- | --- |
| **DESIRABLE EFFECTS** | Trivial | Small | **Moderate** | Large |  | Varies | Don't know |
| **UNDESIRABLE EFFECTS** | Large | Moderate | **Small** | Trivial |  | Varies | Don't know |
| **CERTAINTY OF EVIDENCE** | **Very low** | Low | Moderate | High |  |  | No included studies |
| **VALUES** | Important uncertainty or variability | **Possibly important uncertainty or variability** | Probably no important uncertainty or variability | No important uncertainty or variability |  |  |  |
| **BALANCE OF EFFECTS** | Favors the comparison | Probably favors the comparison | Does not favor either the intervention or the comparison | **Probably favors the intervention** | Favors the intervention | Varies | Don't know |
| **RESOURCES REQUIRED** | Large costs | Moderate costs | **Negligible costs and savings** | Moderate savings | Large savings | Varies | Don't know |
| **COST EFFECTIVENESS** | Favors the comparison | Probably favors the comparison | Does not favor either the intervention or the comparison | Probably favors the intervention | Favors the intervention | Varies | **No included studies** |
| **EQUITY** | Reduced | Probably reduced | **Probably no impact** | Probably increased | Increased | Varies | Don't know |
| **ACCEPTABILITY** | No | Probably no | **Probably yes** | Yes |  | Varies | Don't know |
| **FEASIBILITY** | No | Probably no | **Probably yes** | Yes |  | Varies | Don't know |

# TYPE OF RECOMMENDATION

| Strong recommendation against the intervention | Conditional recommendation against the intervention | Conditional recommendation for either the intervention or the comparison | **Conditional recommendation for the intervention** | Strong recommendation for the intervention |
| --- | --- | --- | --- | --- |
| ○ | ○ | ○ | **●** | ○ |

# CONCLUSIONS

| Recommendation |
| --- |
| The KSA panel suggests initiating a csDMARD with short-term (< 3 months) glucocorticoids over initiating a csDMARD without short-term glucocorticoids in DMARD-naive patients with moderate-to-  high disease activity (conditional recommendation, based on very low certainty evidence)  Remarks:   - The physician should ensure clear communication to the patient of the potential benefits and harms of glucocorticoids - The choice should account for the patient's views on the expected benefits and harms of glucocorticoids - A glucocorticoid treatment regimen should use the lowest dose of for the shortest period possible to reduce harms, with particular caution in the elderly - It is important to monitor for the side effects of glucocorticoids |
|  |
| Justification |
|  |

| Subgroup considerations |
| --- |
|  |
| Implementation considerations |
|  |

| Monitoring and evaluation |
| --- |
|  |
| Research priorities |
|  |

| QUESTION | |
| --- | --- |
| **Should switching to subcutaneous methotrexate vs. addition/switching to alternative DMARD(s) be used for patients taking oral methotrexate who are not at target?** | |
| **POPULATION:** | patients taking oral methotrexate who are not at target |
| **INTERVENTION:** | switching to subcutaneous methotrexate |
| **COMPARISON:** | addition/switching to alternative DMARD(s) |
| **MAIN OUTCOMES:** |  |
| **SETTING:** |  |
| **PERSPECTIVE:** |  |
| **BACKGROUND:** |  |
| **CONFLICT OF INTERESTS:** |  |

# ASSESSMENT

| Desirable Effects How substantial are the desirable anticipated effects? | | |
| --- | --- | --- |
| JUDGEMENT | RESEARCH EVIDENCE | ADDITIONAL CONSIDERATIONS |
| ○ Trivial ● Small ○ Moderate ○ Large ○ Varies ○ Don't know | There are data for only one comparison of switching to subcutaneous MTX versus increasing the dose of oral MTX (indirectly relevant data).  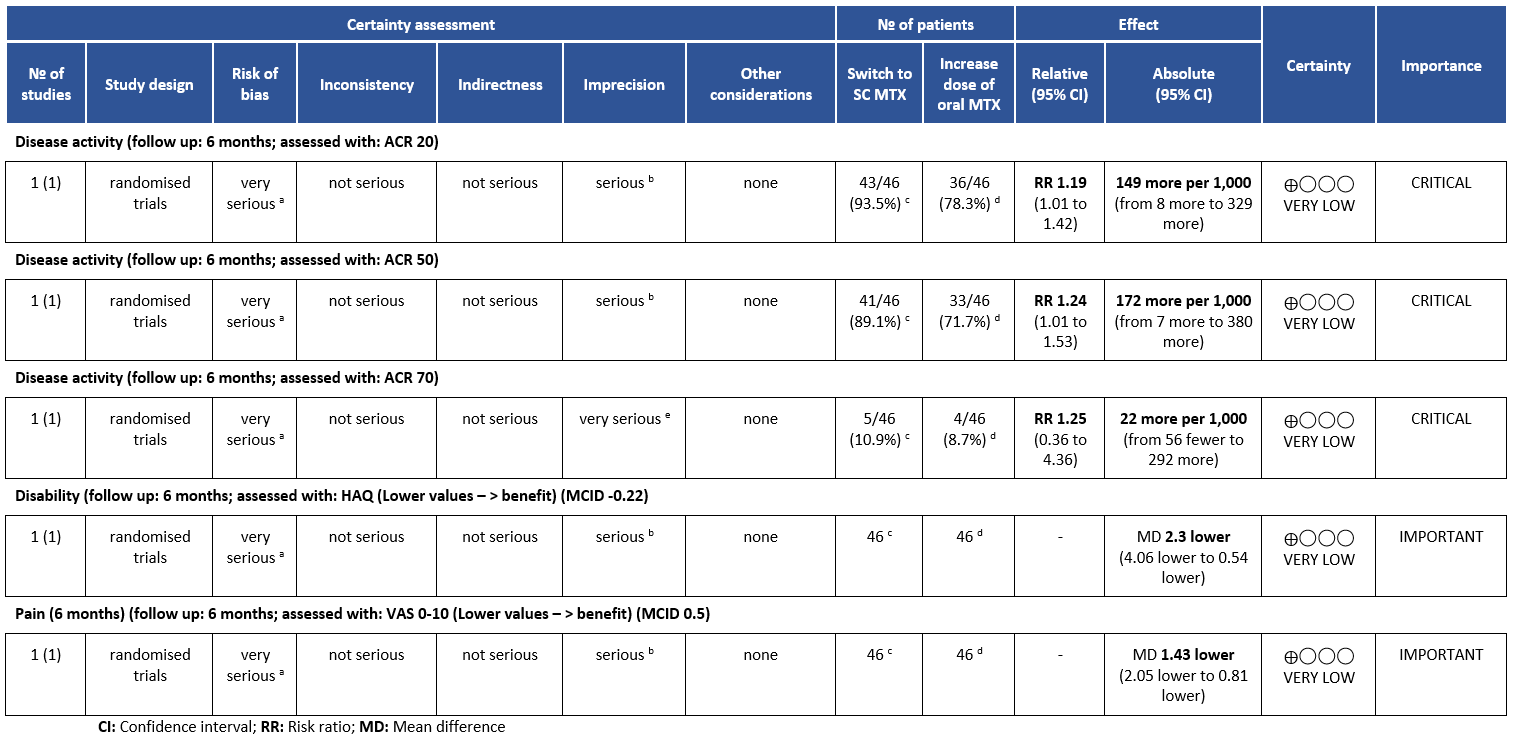  **Explanations**   1. Downgraded by two levels due to very serious risk of bias. Lack of blinding and lack of allocation concealment. 2. Downgraded by one level due to serious imprecision. Very small sample size. 3. SC 20mg MTX for 4 weeks, then 25mg MTX for 8 weeks. 4. PO 20mg MTX for 4 weeks, then 25mg MTX for 8 weeks. 5. e. Downgraded by two levels due to very serious imprecision. Confidence interval includes both values suggesting benefit and values suggesting harm. Very small sample size. | - Most patients eligible to this recommendation are likely to have moderate or high disease activity, but some patients could have low disease activity - Efficacy depends on the use of an optimal dose of SC MTX - Judgment affected by the uncertainty (very low certainty evidence)   Trivial: 1/17 (6%)  Small: 12/17 (71%)  Moderate: 4/17 (24%)  Large: 0/17 (0%)  Varies: 0/17 (0%)  Don't know: 0/17 (0%) |
| Undesirable Effects How substantial are the undesirable anticipated effects? | | |
| JUDGEMENT | RESEARCH EVIDENCE | ADDITIONAL CONSIDERATIONS |
| ○ Large ○ Moderate ● Small ○ Trivial ○ Varies ○ Don't know | No evidence identified. | Large: 0/16 (0%)  Moderate: 3/16 (19%)  Small: 10/16 (63%)  Trivial: 3/16 (19%)  Varies: 0/16 (0%)  Don't know: 0/16 (0%) |
| Certainty of evidence What is the overall certainty of the evidence of effects? | | |
| JUDGEMENT | RESEARCH EVIDENCE | ADDITIONAL CONSIDERATIONS |
| ● Very low ○ Low ○ Moderate ○ High ○ No included studies |  |  |
| Values Is there important uncertainty about or variability in how much people value the main outcomes? | | |
| JUDGEMENT | RESEARCH EVIDENCE | ADDITIONAL CONSIDERATIONS |
| ○ Important uncertainty or variability ● Possibly important uncertainty or variability ○ Probably no important uncertainty or variability ○ No important uncertainty or variability | • Patient preferences are variable  • Treatment benefit is more important than non-serious and serious adverse events  • Treatment benefit is more important than route of administration  • Route of administration is often more important than serious/non-serious adverse events | Important uncertainty or variability: 2/16 (13%)  Possibly important uncertainty or variability: 11/16 (69%)  Probably no important uncertainty or variability: 3/16 (19%)  No important uncertainty or variability: 0/16 (0%) |
| Balance of effects Does the balance between desirable and undesirable effects favor the intervention or the comparison? | | |
| JUDGEMENT | RESEARCH EVIDENCE | ADDITIONAL CONSIDERATIONS |
| ○ Favors the comparison ○ Probably favors the comparison ○ Does not favor either the intervention or the comparison ● Probably favors the intervention ○ Favors the intervention ○ Varies ○ Don't know |  | Favors the comparison: 0/16 (0%)  Probably favors the comparison: 0/16 (0%)  Does not favor either the intervention or the comparison: 1/16 (6%)  Probably favors the intervention: 14/16 (88%)  Favors the intervention: 1/16 (6%)  Varies: 0/16 (0%)  Don't know: 0/16 (0%) |
| Resources required How large are the resource requirements (costs)? | | |
| JUDGEMENT | RESEARCH EVIDENCE | ADDITIONAL CONSIDERATIONS |
| ○ Large costs ● Moderate costs ○ Negligible costs and savings ○ Moderate savings ○ Large savings ○ Varies ○ Don't know | 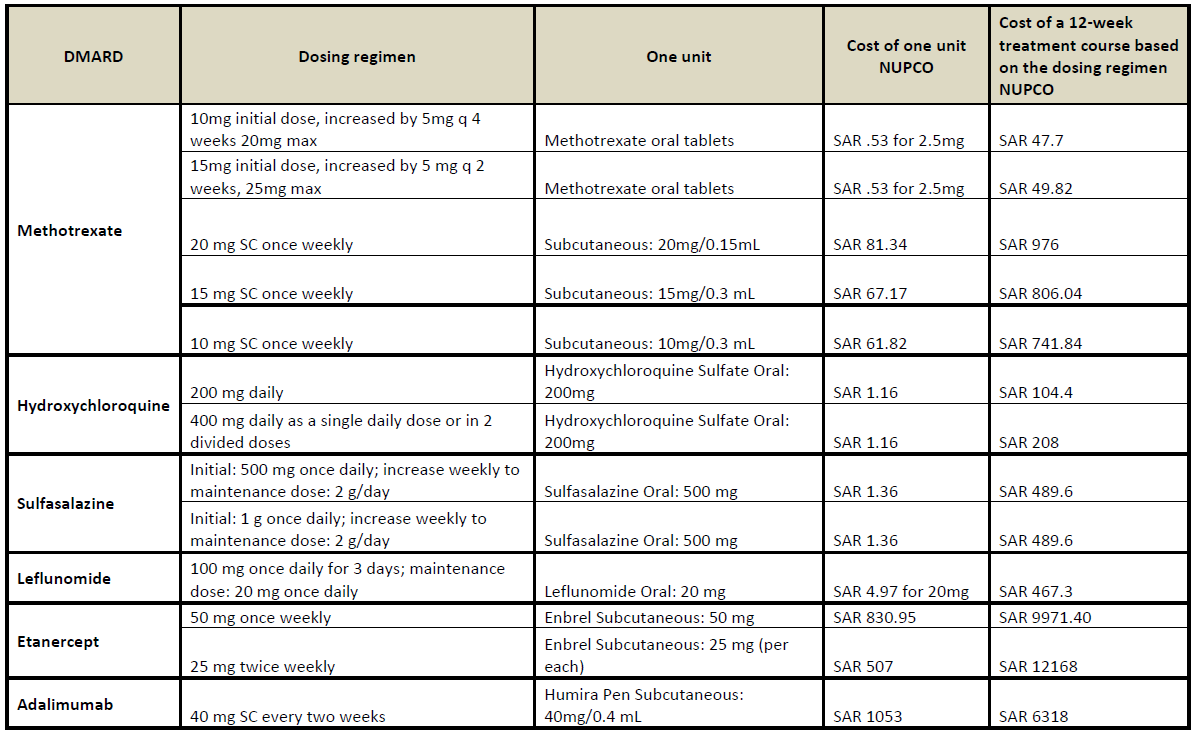  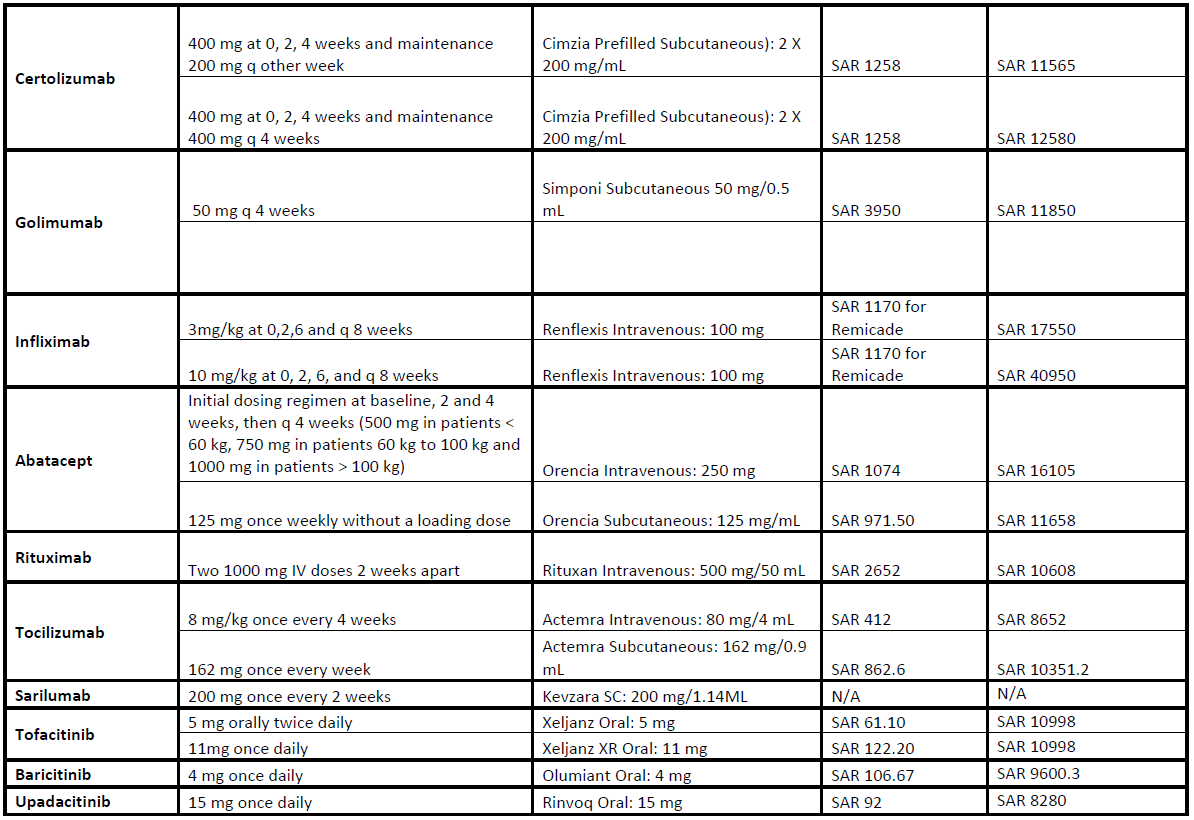 | Large costs: 0/16 (0%)  Moderate costs: 11/16 (69%)  Negligible costs and savings: 4/16 (25%)  Moderate savings: 1/16 (6%)  Large savings: 0/16 (0%)  Varies: 0/16 (0%)  Don't know: 0/16 (0%) |
| Cost effectiveness Does the cost-effectiveness of the intervention favor the intervention or the comparison? | | |
| JUDGEMENT | RESEARCH EVIDENCE | ADDITIONAL CONSIDERATIONS |
| ○ Favors the comparison ○ Probably favors the comparison ○ Does not favor either the intervention or the comparison ○ Probably favors the intervention ○ Favors the intervention ○ Varies ● No included studies |  | Favors the comparison: 0/16 (0%)  Probably favors the comparison: 2/16 (13%)  Does not favor either the intervention or the comparison: 2/16 (13%)  Probably favors the intervention: 1/16 (6%)  Favors the intervention: 0/16 (0%)  Varies: 0/16 (0%)  No included studies: 11/16 (69%) |
| Equity What would be the impact on health equity? | | |
| JUDGEMENT | RESEARCH EVIDENCE | ADDITIONAL CONSIDERATIONS |
| ○ Reduced ○ Probably reduced ○ Probably no impact ○ Probably increased ○ Increased ● Varies ○ Don't know |  | - Currently, MTX SC not widely available (e.g., in private hospitals some governmental hospitals), so might negatively affect health equity. - A recommendation in favor of SC MTX would improve equity by increasing availability - Minorities (e.g., expats) are typically on private insurance with whom coverage might vary.   Reduced: 0/15 (0%)  Probably reduced: 6/15 (40%)  Probably no impact: 0/15 (0%)  Probably increased: 5/15 (33%)  Increased: 0/15 (0%)  Varies: 4/15 (27%)  Don't know: 0/15 (0%) |
| Acceptability Is the intervention acceptable to key stakeholders? | | |
| JUDGEMENT | RESEARCH EVIDENCE | ADDITIONAL CONSIDERATIONS |
| ○ No ○ Probably no ● Probably yes ○ Yes ○ Varies ○ Don't know |  | No: 0/15 (0%)  Probably no: 1/15 (7%)  Probably yes: 13/15 (87%)  Yes: 1/15 (7%)  Varies: 0/15 (0%)  Don't know: 0/15 (0%) |
| Feasibility Is the intervention feasible to implement? | | |
| JUDGEMENT | RESEARCH EVIDENCE | ADDITIONAL CONSIDERATIONS |
| ○ No ○ Probably no ● Probably yes ○ Yes ○ Varies ○ Don't know |  | - Issues related to availability as noted above - Prefilled is not available in SC, 15mg is only available as vial   No: 0/14 (0%)  Probably no: 0/14 (0%)  Probably yes: 12/14 (86%)  Yes: 0/14 (0%)  Varies: 2/14 (14%)  Don't know: 0/14 (0%) |

# SUMMARY OF JUDGEMENTS

|  | **JUDGEMENT** | | | | | | |
| --- | --- | --- | --- | --- | --- | --- | --- |
| **DESIRABLE EFFECTS** | Trivial | **Small** | Moderate | Large |  | Varies | Don't know |
| **UNDESIRABLE EFFECTS** | Large | Moderate | **Small** | Trivial |  | Varies | Don't know |
| **CERTAINTY OF EVIDENCE** | **Very low** | Low | Moderate | High |  |  | No included studies |
| **VALUES** | Important uncertainty or variability | **Possibly important uncertainty or variability** | Probably no important uncertainty or variability | No important uncertainty or variability |  |  |  |
| **BALANCE OF EFFECTS** | Favors the comparison | Probably favors the comparison | Does not favor either the intervention or the comparison | **Probably favors the intervention** | Favors the intervention | Varies | Don't know |
| **RESOURCES REQUIRED** | Large costs | **Moderate costs** | Negligible costs and savings | Moderate savings | Large savings | Varies | Don't know |
| **COST EFFECTIVENESS** | Favors the comparison | Probably favors the comparison | Does not favor either the intervention or the comparison | Probably favors the intervention | Favors the intervention | Varies | **No included studies** |
| **EQUITY** | Reduced | Probably reduced | Probably no impact | Probably increased | Increased | **Varies** | Don't know |
| **ACCEPTABILITY** | No | Probably no | **Probably yes** | Yes |  | Varies | Don't know |
| **FEASIBILITY** | No | Probably no | **Probably yes** | Yes |  | Varies | Don't know |

# TYPE OF RECOMMENDATION

| Strong recommendation against the intervention | Conditional recommendation against the intervention | Conditional recommendation for either the intervention or the comparison | **Conditional recommendation for the intervention** | Strong recommendation for the intervention |
| --- | --- | --- | --- | --- |
| ○ | ○ | ○ | **●** | ○ |

# CONCLUSIONS

| Recommendation |
| --- |
| The KSA panel suggests a switch to subcutaneous methotrexate over addition/switch to alternative DMARD(s) in patients taking oral methotrexate who are not at target (conditional recommendation, based on very low certainty evidence)  Remarks:   - This recommendation typically applies to patients with moderate or high disease activity, but may apply to patients with low disease activity - Target may differ based on initial disease activity status. Target can be low in patients who desire remission - The choice should consider the availability of the medications - It is important to monitor the medications' side effects and adjust regimen accordingly |
|  |
| Justification |
|  |

| Subgroup considerations |
| --- |
|  |
| Implementation considerations |
|  |

| Monitoring and evaluation | |
| --- | --- |
|  | |
| Research priorities | |
|  | |
| QUESTION | |
| **Should gradual discontinuation of methotrexate vs. gradual discontinuation of the bDMARD or tsDMARD be used for patients taking methotrexate plus a bDMARD or tsDMARD who wish to discontinue a DMARD?** | |
| **POPULATION:** | patients taking methotrexate plus a bDMARD or tsDMARD who wish to discontinue a DMARD |
| **INTERVENTION:** | gradual discontinuation of methotrexate |
| **COMPARISON:** | gradual discontinuation of the bDMARD or tsDMARD |
| **MAIN OUTCOMES:** | Disease-worsening; Serious adverse events (AEs); |
| **SETTING:** |  |
| **PERSPECTIVE:** |  |
| **BACKGROUND:** |  |
| **CONFLICT OF INTERESTS:** |  |

# ASSESSMENT

| Desirable Effects How substantial are the desirable anticipated effects? | | |
| --- | --- | --- |
| JUDGEMENT | RESEARCH EVIDENCE | ADDITIONAL CONSIDERATIONS |
| ○ Trivial ○ Small ● Moderate ○ Large ○ Varies ○ Don't know | \| **Outcomes** \| **№ of participants (studies) Follow-up** \| **Certainty of the evidence (GRADE)** \| **Relative effect (95% CI)** \| **Anticipated absolute effects^*^ (95% CI)** \| \| \| --- \| --- \| --- \| --- \| --- \| --- \| \| **Risk with gradual discontinuation of the bDMARD or tsDMARD** \| **Risk difference with gradual discontinuation of methotrexate** \| \| Disease-worsening follow-up: 48 weeks \| 202 (1 RCT) \| ⨁⨁⨁◯ Moderate^a,b^ \| **RR 0.63** (0.48 to 0.84) \| Study population \| \| \| 624 per 1,000 \| **231 fewer per 1,000** (324 fewer to 100 fewer) \|   **Explanations**   1. Downgraded by one level for risk of bias due to missing outcome data (9/101 participants in the MTX discontinuation arm and 13/101 participants in the Etanercept discontinuation arm) and imprecision due to a relatively small number of events. 2. bDMARDs include ETN | Trivial: 0/15 (0%)  Small: 3/15 (20%)  Moderate: 8/15 (53%)  Large: 3/15 (20%)  Varies: 0/15 (0%)  Don't know: 1/15 (7%) |
| Undesirable Effects How substantial are the undesirable anticipated effects? | | |
| JUDGEMENT | RESEARCH EVIDENCE | ADDITIONAL CONSIDERATIONS |
| ○ Large ○ Moderate ○ Small ● Trivial ○ Varies ○ Don't know | \| **Outcomes** \| **№ of participants (studies) Follow-up** \| **Certainty of the evidence (GRADE)** \| **Relative effect (95% CI)** \| **Anticipated absolute effects^*^ (95% CI)** \| \| \| --- \| --- \| --- \| --- \| --- \| --- \| \| **Risk with gradual discontinuation of the bDMARD or tsDMARD** \| **Risk difference with gradual discontinuation of methotrexate** \| \| Serious adverse events (AEs) \| 199 (1 RCT) \| ⨁⨁◯◯ Low^a,b,c^ \| **RR 1.01** (0.25 to 4.16) \| Study population \| \| \| 40 per 1,000 \| **0 fewer per 1,000** (30 fewer to 126 more) \|   **Explanations**   1. Downgraded by one level for imprecision due to a relatively small number of events. 2. Downgraded by one level for risk of bias due to missing outcome data (9/101 participants in the MTX discontinuation arm and 13/101 participants in the Etanercept discontinuation arm). 3. bDMARDs include ETN | Large: 0/15 (0%)  Moderate: 4/15 (27%)  Small: 5/15 (33%)  Trivial: 6/15 (40%)  Varies: 0/15 (0%)  Don't know: 0/15 (0%) |
| Certainty of evidence What is the overall certainty of the evidence of effects? | | |
| JUDGEMENT | RESEARCH EVIDENCE | ADDITIONAL CONSIDERATIONS |
| ○ Very low ○ Low ● Moderate ○ High ○ No included studies |  |  |
| Values Is there important uncertainty about or variability in how much people value the main outcomes? | | |
| JUDGEMENT | RESEARCH EVIDENCE | ADDITIONAL CONSIDERATIONS |
| ○ Important uncertainty or variability ● Possibly important uncertainty or variability ○ Probably no important uncertainty or variability ○ No important uncertainty or variability | • Patient preferences are variable  • Treatment benefit is more important than non-serious and serious adverse events  • Treatment benefit is more important than route of administration  • Route of administration is often more important than serious/non-serious adverse events | Important uncertainty or variability: 2/16 (13%)  Possibly important uncertainty or variability: 11/16 (69%)  Probably no important uncertainty or variability: 3/16 (19%)  No important uncertainty or variability: 0/16 (0%) |
| Balance of effects Does the balance between desirable and undesirable effects favor the intervention or the comparison? | | |
| JUDGEMENT | RESEARCH EVIDENCE | ADDITIONAL CONSIDERATIONS |
| ○ Favors the comparison ○ Probably favors the comparison ○ Does not favor either the intervention or the comparison ● Probably favors the intervention ○ Favors the intervention ○ Varies ○ Don't know |  | Favors the comparison: 0/15 (0%)  Probably favors the comparison: 2/15 (13%)  Does not favor either the intervention or the comparison: 0/15 (0%)  Probably favors the intervention: 11/15 (73%)  Favors the intervention: 2/15 (13%)  Varies: 0/15 (0%)  Don't know: 0/15 (0%) |
| Resources required How large are the resource requirements (costs)? | | |
| JUDGEMENT | RESEARCH EVIDENCE | ADDITIONAL CONSIDERATIONS |
| ○ Large costs ● Moderate costs ○ Negligible costs and savings ○ Moderate savings ○ Large savings ○ Varies ○ Don't know | 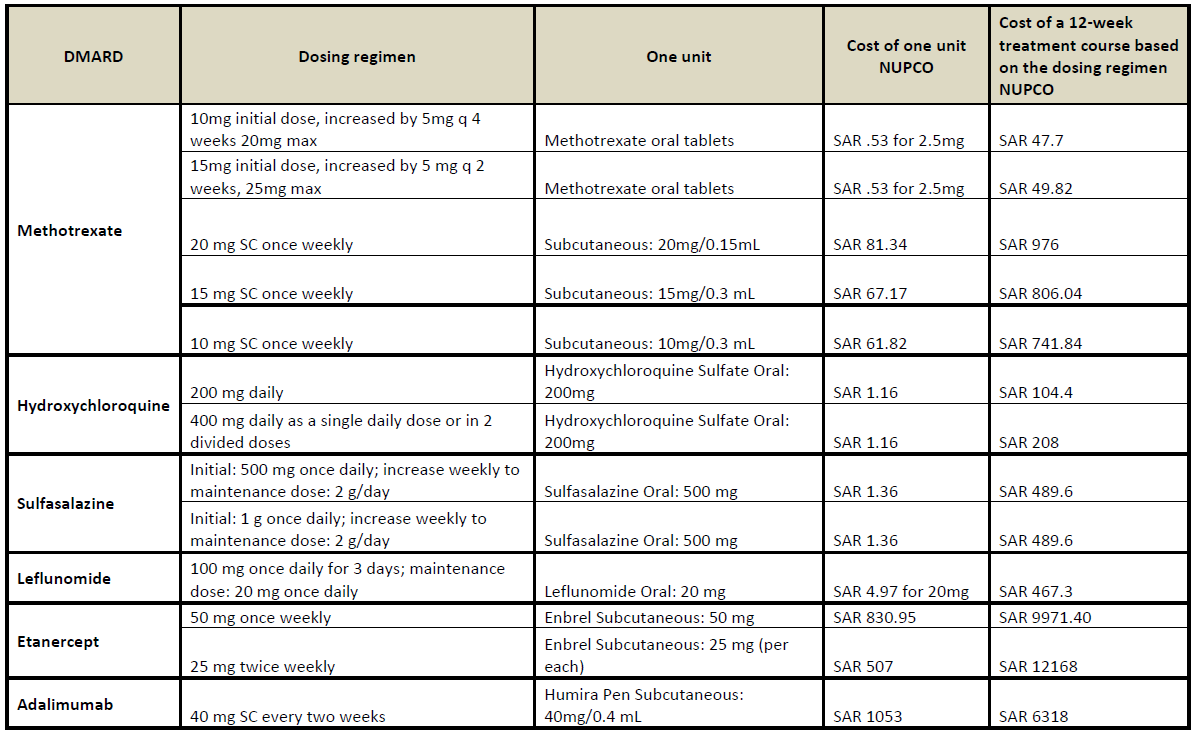  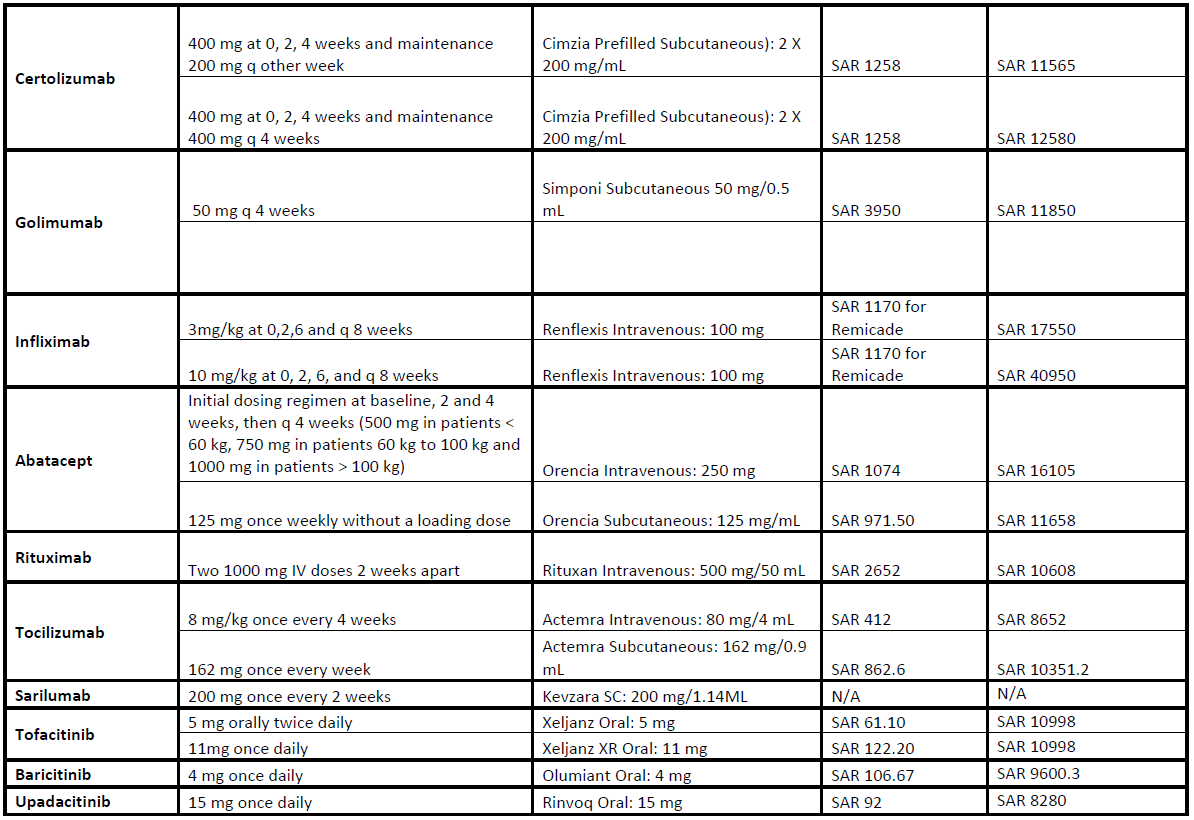 | - Several biosimilars have been approved by Saudi FDA. - Prices for biosimilars are significantly reduced compared with prices listed here (about 30% lower). That would reduce overall costs and would increase accessibility. - ACR background: Biosimilars are considered equivalent to FDA-approved originator bDMARDs.   Large costs: 1/16 (6%)  Moderate costs: 9/16 (56%)  Negligible costs and savings: 0/16 (0%)  Moderate savings: 4/16 (25%)  Large savings: 0/16 (0%)  Varies: 2/16 (13%)  Don't know: 0/16 (0%) |
| Cost effectiveness Does the cost-effectiveness of the intervention favor the intervention or the comparison? | | |
| JUDGEMENT | RESEARCH EVIDENCE | ADDITIONAL CONSIDERATIONS |
| ○ Favors the comparison ○ Probably favors the comparison ○ Does not favor either the intervention or the comparison ○ Probably favors the intervention ○ Favors the intervention ○ Varies ● No included studies |  | Favors the comparison: 1/16 (6%)  Probably favors the comparison: 2/16 (13%)  Does not favor either the intervention or the comparison: 0/16 (0%)  Probably favors the intervention: 3/16 (19%)  Favors the intervention: 0/16 (0%)  Varies: 0/16 (0%)  No included studies: 10/16 (63%) |
| Equity What would be the impact on health equity? | | |
| JUDGEMENT | RESEARCH EVIDENCE | ADDITIONAL CONSIDERATIONS |
| ○ Reduced ○ Probably reduced ○ Probably no impact ○ Probably increased ○ Increased ● Varies ○ Don't know |  | - Patients already on 2 medications, not clear this would affect equity. - Stopping a medication would make it more available to others. - There are no out of pocket payments in the KSA setting; focus is on value based care   Reduced: 1/15 (7%)  Probably reduced: 4/15 (27%)  Probably no impact: 3/15 (20%)  Probably increased: 4/15 (27%)  Increased: 0/15 (0%)  Varies: 1/15 (7%)  Don't know: 2/15 (13%) |
| Acceptability Is the intervention acceptable to key stakeholders? | | |
| JUDGEMENT | RESEARCH EVIDENCE | ADDITIONAL CONSIDERATIONS |
| ○ No ○ Probably no ● Probably yes ○ Yes ○ Varies ○ Don't know |  | No: 0/15 (0%)  Probably no: 1/15 (7%)  Probably yes: 13/15 (87%)  Yes: 1/15 (7%)  Varies: 0/15 (0%)  Don't know: 0/15 (0%) |
| Feasibility Is the intervention feasible to implement? | | |
| JUDGEMENT | RESEARCH EVIDENCE | ADDITIONAL CONSIDERATIONS |
| ○ No ○ Probably no ● Probably yes ○ Yes ○ Varies ○ Don't know |  | No: 0/14 (0%)  Probably no: 0/14 (0%)  Probably yes: 14/14 (100%)  Yes: 0/14 (0%)  Varies: 0/14 (0%)  Don't know: 0/14 (0%) |

# SUMMARY OF JUDGEMENTS

|  | **JUDGEMENT** | | | | | | |
| --- | --- | --- | --- | --- | --- | --- | --- |
| **DESIRABLE EFFECTS** | Trivial | Small | **Moderate** | Large |  | Varies | Don't know |
| **UNDESIRABLE EFFECTS** | Large | Moderate | Small | **Trivial** |  | Varies | Don't know |
| **CERTAINTY OF EVIDENCE** | Very low | Low | **Moderate** | High |  |  | No included studies |
| **VALUES** | Important uncertainty or variability | **Possibly important uncertainty or variability** | Probably no important uncertainty or variability | No important uncertainty or variability |  |  |  |
| **BALANCE OF EFFECTS** | Favors the comparison | Probably favors the comparison | Does not favor either the intervention or the comparison | **Probably favors the intervention** | Favors the intervention | Varies | Don't know |
| **RESOURCES REQUIRED** | Large costs | **Moderate costs** | Negligible costs and savings | Moderate savings | Large savings | Varies | Don't know |
| **COST EFFECTIVENESS** | Favors the comparison | Probably favors the comparison | Does not favor either the intervention or the comparison | Probably favors the intervention | Favors the intervention | Varies | **No included studies** |
| **EQUITY** | Reduced | Probably reduced | Probably no impact | Probably increased | Increased | **Varies** | Don't know |
| **ACCEPTABILITY** | No | Probably no | **Probably yes** | Yes |  | Varies | Don't know |
| **FEASIBILITY** | No | Probably no | **Probably yes** | Yes |  | Varies | Don't know |

# TYPE OF RECOMMENDATION

| Strong recommendation against the intervention | Conditional recommendation against the intervention | Conditional recommendation for either the intervention or the comparison | **Conditional recommendation for the intervention** | Strong recommendation for the intervention |
| --- | --- | --- | --- | --- |
| ○ | ○ | ○ | **●** | ○ |

# CONCLUSIONS

| Recommendation |
| --- |
| The KSA panel suggests gradual discontinuation of methotrexate over gradual discontinuation of the bDMARD or tsDMARD for patients taking methotrexate plus a bDMARD or tsDMARD who wish to discontinue a DMARD (conditional recommenadtion;, based on low certainty evidence)  Remarks:   - The patient and the physician should closely monitor the progression of symptoms during and following discontinuation |
|  |
| Justification |
|  |

| Subgroup considerations |
| --- |
|  |
| Implementation considerations |
|  |

| Monitoring and evaluation |
| --- |
|  |
| Research priorities |
